# An N-acetyltransferase required for ESAT-6 N-terminal acetylation and virulence in *Mycobacterium marinum*

**DOI:** 10.1128/mbio.00987-23

**Published:** 2023-09-29

**Authors:** Owen A. Collars, Bradley S. Jones, Daniel D. Hu, Simon D. Weaver, Taylor A. Sherman, Matthew M. Champion, Patricia A. Champion

**Affiliations:** 1 Department of Biological Sciences, University of Notre Dame, Notre Dame, Indiana, USA; 2 Eck Institute for Global Health, University of Note Dame, Notre Dame, Indiana, USA; 3 Department of Chemistry and Biochemistry, University of Notre Dame, Notre Dame, Indiana, USA; Weill Cornell Medicine, New York, New York, USA

**Keywords:** ESAT-6, acetylation, N-acetyltransferase, *Mycobacterium*, ESX-1

## Abstract

N-terminal protein acetylation is a ubiquitous post-translational modification that impacts diverse cellular processes in higher organisms. Bacterial proteins are also N-terminally acetylated, but the mechanisms and consequences of this modification in bacteria are poorly understood. The major virulence factor EsxA (ESAT-6, early secreted antigen, 6 kDa) was one of the first N-terminally acetylated proteins identified in bacteria. EsxA is conserved in mycobacterial pathogens, including *Mycobacterium tuberculosis* and *Mycobacterium marinum*, a non-tubercular mycobacterial species that causes tuberculosis-like disease in ectotherms. However, the enzyme responsible for EsxA N-terminal acetylation has been elusive. Here, we used genetics, molecular biology, and mass-spectroscopy-based proteomics to demonstrate that MMAR_1839 (renamed Emp1, ESX-1 modifying protein, 1) is the putative N-acetyltransferase (NAT) solely responsible for EsxA acetylation in *M. marinum*. We demonstrated that *ERD_3144*, the orthologous gene in *M. tuberculosis* Erdman, is functionally equivalent to Emp1. We previously quantified widespread N-terminal protein acetylation in pathogenic mycobacteria (C. R. Thompson, M. M. Champion, and P. A. Champion, J Proteome Res 17:3246–3258, 2018, https:// doi: 10.1021/acs.jproteome.8b00373). We identified at least 22 additional proteins that require Emp1 for acetylation, demonstrating that this putative NAT is not dedicated to EsxA. Finally, we showed that loss of *emp1* did not prevent phagosomal escape but resulted in a significant reduction in macrophage cytolysis by and cell-to-cell spread of *M. marinum* during infection. Collectively, this study identified a NAT required for N-terminal acetylation and pathogenesis in *Mycobacterium*.

**IMPORTANCE:**

N-terminal acetylation is a protein modification that broadly impacts basic cellular function and disease in higher organisms. Although bacterial proteins are N-terminally acetylated, little is understood how N-terminal acetylation impacts bacterial physiology and pathogenesis. Mycobacterial pathogens cause acute and chronic disease in humans and in animals. Approximately 15% of mycobacterial proteins are N-terminally acetylated, but the responsible enzymes are largely unknown. We identified a conserved mycobacterial protein required for the N-terminal acetylation of 23 mycobacterial proteins including the EsxA virulence factor. Loss of this enzyme from *M. marinum* reduced macrophage killing and spread of *M. marinum* to new host cells. Defining the acetyltransferases responsible for the N-terminal protein acetylation of essential virulence factors could lead to new targets for therapeutics against mycobacteria.

## INTRODUCTION

EsxA (ESAT-6) is a major mycobacterial virulence factor required for the pathogenesis of *Mycobacterium tuberculosis* and other mycobacterial pathogens (1 – 4). EsxA is secreted by the ESX-1 (ESAT-6 system-1) protein secretion system and is required for the secretion of the majority of the ESX-1 substrates (5 – 7). Early during macrophage infection, ESX-1 substrates are required for damaging the phagosomal membrane (4, 6, 8 – 10), allowing mycobacterial pathogens to access the macrophage cytoplasm (11, 12). Exposure to the cytoplasm allows *Mycobacterium* to combat the host response and causes macrophage cytolysis which promotes mycobacterial spread (9, 13 – 17). *Mycobacterium* lacking the ESX-1 system are retained in the phagosome and attenuated (11). EsxA may play additional roles in the host downstream of phagosomal lysis (18 – 24).

N-terminal acetylation is the covalent addition of an acetyl group to the α amino group of the N-terminal amino acid of a protein by N-acetyltransferases (NATs) (25 – 27). NATs can irreversibly acetylate the iMet (initiator Met, following deformylation) or the first amino acid following iMet cleavage (28, 29). EsxA was one of the first bacterial proteins recognized to be N-terminally acetylated (30). The role of EsxA N-terminal acetylation has been linked to protein interaction *in vitro* (30, 31), and phagosomal lysis (31) using *Mycobacterium marinum*, an established model for studying the mycobacterial ESX-1 system (32). Importantly, the deletion of *esx-1* genes in *M. marinum* is functionally complemented by the expression of orthologous genes from *M. tuberculosis*, demonstrating a conserved function (33).

In higher organisms, including humans, yeasts, and plants, ~65%–85% of proteins are N-terminally acetylated by seven NATs (25). In these organisms, N-terminal acetylation directly impacts protein function (25, 34 – 42). ~10%–15% of bacterial proteins are estimated to be N-terminally acetylated (26, 43 – 46). Using quantitative N-terminomics, we observed that ~11% and ~15% of proteins in *M. tuberculosis* and *M. marinum*, respectively, are N-terminally acetylated during standard laboratory growth (43). We found that several additional ESX-1 substrates and at least one ESX-1 membrane component are also N-terminally acetylated (43).

Bacterial genomes encode several putative NATs, which are part of the GNAT (GCN-5-related NAT) family (26, 47). It is impossible to predict if the putative NAT acetylates Lys residues (KATs), small molecules, antibiotics, and/or protein N-termini (26). There are 27 predicted NATs in *M. tuberculosis*, 23 of which are conserved in *M. marinum* (43). The best characterized NAT is RimI, which N-terminally acetylates the S18 rRNA protein and functions as a generalist KAT in *Escherichia coli*, *Salmonella*, and in *Mycobacterium* (47 – 51). The individual NATs responsible for N-terminal acetylation of specific mycobacterial proteins are unknown, limiting our understanding of how this modification impacts virulence and physiology.

The EsxA NAT or NATs have remained elusive. Changes in EsxA acetylation are difficult to assess because the amino acid composition of EsxA yields a large N-terminal tryptic fragment with poor chromatographic performance by mass-spectroscopy-based proteomics (52 – 54). Top-down approaches do not rapidly assign all N-terminal isoforms of EsxA (52 – 54).

We sought to identify the NAT responsible for N-terminally acetylating EsxA to further understand N-terminal acetylation in *Mycobacterium*. Based on the conservation of EsxA and the putative NATs between *M. marinum* and *M. tuberculosis*, we hypothesized that we could leverage the use of *M. marinum* to identify the conserved EsxA NAT. To test this hypothesis, we used an N-terminal acetyl-EsxA antibody coupled with a knockout *M. marinum* strain collection to identify the EsxA NAT. We measured EsxA acetylation in the presence and absence of the putative NAT using western blot analysis, MALDI, and label-free quantitative (LFQ) mass spectrometry. We tested ESX-1 function and mycobacterial virulence using *in vitro* systems and a macrophage model of infection.

## RESULTS

### *In vitro* discrimination between the EsxA and acetyl-EsxA N-termini

**Fig 1:**
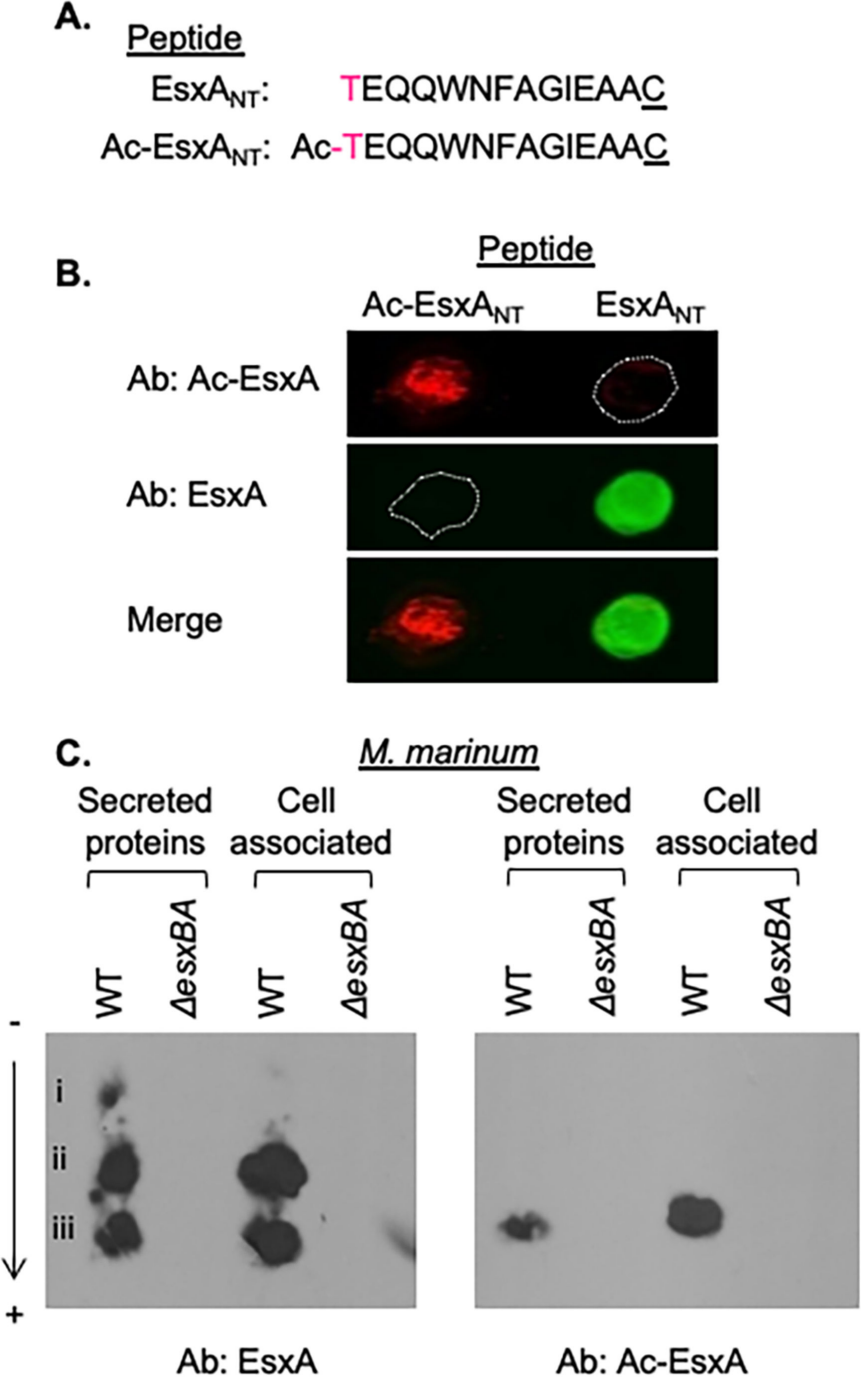
The Ac-EsxA antibody specifically recognizes Ac-EsxA in complex mixtures. (A) The EsxA and Ac-EsxA N-terminal peptides. The “C” is not native to the EsxA protein. NT subscript = N-terminal. (B) Dot blot of EsxA N-terminal peptides. Twenty micrograms of each peptide was spotted on nitrocellulose and immunoblotted with the ⍺EsxA and ⍺Ac-EsxA antibodies. The image is representative of three independent replicates. (C) NUT-PAGE of secreted and cell-associated proteins from WT and Δ*esxBA M. marinum* strains. Twenty micrograms of protein was loaded in each lane. The image is representative of at least three independent biological replicates.

We obtained and characterized a polyclonal antibody synthesized against an acetylated N-terminal EsxA peptide (Fig. 1A, Ac-EsxA_NT_) for use as a tool to identify the NAT(s) responsible for N-terminal acetylation of EsxA. We performed a dot blot to determine if the Ac-EsxA antibody specifically recognized the acetylated N-terminus of EsxA. The Ac-EsxA antibody specifically produced a signal where the Ac-EsxA_NT_ peptide was spotted on the nitrocellulose (red) but not where the unacetylated peptide was spotted (Fig. 1B, EsxA_NT_, outline). In contrast, a commercially available EsxA antibody raised against EsxA_NT_ peptide specifically produced a signal where the unacetylated EsxA peptide was spotted onto the nitrocellulose (green), but not where the Ac-EsxA_NT_ peptide was spotted. From these data, we conclude that the Ac-EsxA antibody discriminates between the acetylated and unacetylated forms of the EsxA N-terminal peptide. Moreover, the commercial EsxA antibody specifically recognizes the unacetylated form of EsxA.

We next tested if the Ac-EsxA antibody could detect the acetylated version of the EsxA protein (Ac-EsxA) in a complex mixture. We collected cell-associated and secreted protein fractions from the wild-type (WT) and Δ*esxBA M. marinum* strains. We separated the proteins by charge using neutral pH urea Triton polyacrylamide gel electrophoresis (NUT-PAGE), which separates acetylated and unacetylated proteins (55). NUT-PAGE followed by western blot analysis allowed for the separation of several protein species (i–iii) detected by the EsxA antibody in the protein fractions collected from the WT *M. marinum* strain (Fig. 1C). All three of these species were absent from the protein fractions generated from the Δ*esxBA* strain, which fails to produce EsxA protein. Notably, only species iii, which is the most negatively charged EsxA species, was reliably detected by the Ac-EsxA antibody. From these data, we conclude that we can separate and detect acetylated EsxA from a complex mixture of *M. marinum* proteins.

### N-terminal acetylation of EsxA and other proteins is dependent on MMAR_1839

The EsxA proteins from *M. marinum* and *M. tuberculosis* are identical through the 15th amino acid and 92% identical overall (Fig. S1). Therefore, we reasoned that the NAT responsible for acetylating EsxA would be highly conserved. We identified the five most highly conserved GNAT proteins between the two species (Table 1). We generated unmarked deletions of each putative NAT gene in *M. marinum* and confirmed the deletion of each gene by PCR (Fig. S2) and targeted DNA sequencing.

**TABLE 1:** The top five conserved putative NATs between *M. marinum* and *M. tuberculosis^a^*

*M. marinum* gene	*M. tuberculosis* gene	% identity (protein)
*MMAR_1067*	*Rv0730*	90.9%
*MMAR_1968*	*argA*	87.3%
*MMAR_4519*	*rimJ*	87.53%
*MMAR_1839*	*Rv2867c*	87.32%
*MMAR_1882*	*Rv2851c*	86.75%

^*a*^ Sequences were obtained from Mycobrowser. The percent protein identity was determined using Protein BLAST.

**Fig 2:**
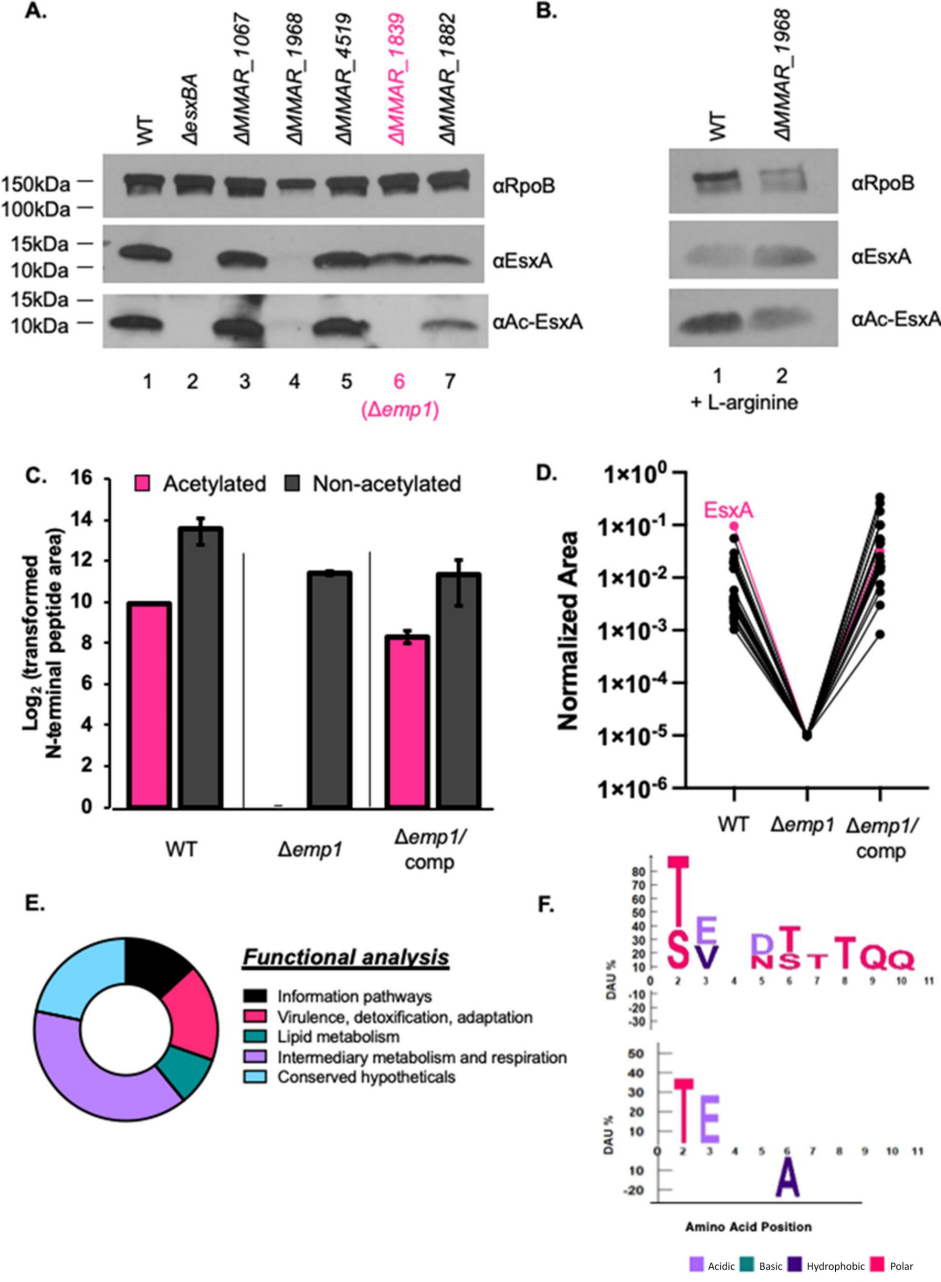
MMAR_1839 (Emp1) is required for the acetylation of EsxA and other proteins. (A and B) Western blot analysis of cell-associated proteins from the indicated *M. marinum* strains. Ten micrograms of protein was loaded in each lane. In panel B, 2 µM of L-arginine was added to the culture media. Both images are representative of at least three biological replicates. RpoB is a control for loading. (C) MS Analysis of relative abundance of acetylated and non-acetylated N-terminus of EsxA. Label-free quantitative (LFQ) proteomics intensity of the EsxA N-terminal peptide from WT, Δ*emp1*, Δ*emp1/*complemented strains. The normalized intensity was transformed by 10^4^ to convert the Log2 values to positive integers. Propagated error was performed on technical triplicates. (D) K-means clustering of all N-terminally acetylated proteins observed from bottom-up proteomics in WT, Δ*emp1*, Δ*emp1/*complemented strains. Shown is the cluster that contained EsxA. (E) Functional analysis from Mycobrowser of the 22 proteins that clustered with EsxA from the k-means analysis from *M. marinum*. For conserved hypothetical proteins, if the closest ortholog in *M. tuberculosis* was annotated, that annotation was used instead. (F) ICE Logo from the protein N-termini in panels D and E. Differential amino acid usage (DAU) tests were used to determine overrepresented and underrepresented amino acids at specific N-terminal amino acid positions (61). Fisher’s exact test with *P* < 0.05 was used to determine significance. The top logo compares the protein N-termini in panels D and E to the whole *M. marinum* proteome; the bottom logo compares them to the *M. marinum* N-terminome from (see supplemental material) reference 43. All R code is available on GitHub (https://github.com/Champion-Lab/ESXA_Acetylation) along with a list of data analysis steps.

We hypothesized that Ac-EsxA would be absent from *M. marinum* strains lacking an EsxA-specific NAT. We collected cell-associated proteins from *M. marinum* strains lacking each of the five most conserved NATs. We measured EsxA and Ac-EsxA in these strains using western blot analysis, as compared to proteins generated from the WT and Δ*esxBA* strains. Both the EsxA and Ac-EsxA proteins were detected in lysates generated from the WT *M. marinum* strain (Fig. 2A, lane 1), consistent with prior studies demonstrating that both species exist in the WT strain (30, 43, 56 – 59). Both EsxA species were lacking from the Δ*esxBA* strain (lane 2), demonstrating the specificity of both antibodies to EsxA. The EsxA and Ac-EsxA proteins were present in the lysates generated from the Δ*MMAR_1067*, Δ*MMAR_4519*, or Δ*MMAR_1882* strains (lanes 3, 5, and 7). The Δ*MMAR_1968* strain lacks the ArgA NAT and is auxotrophic for arginine (60). The Δ*MMAR_1968* strain exhibited reduced growth compared to the WT and complemented strains (Fig. S3). Addition of L-arginine to the growth media restored the growth of the Δ*MMAR_1968* strain (Fig. S3), allowing detection of both Ac-EsxA and EsxA protein from the Δ*MMAR_1968* lysates (Fig. 2B). The EsxA protein (Fig. 2A, lane 6), but not the Ac-EsxA protein, was detected in the lysate from the Δ*MMAR_1839* strain. From these data, we conclude that MMAR_1839 is required for EsxA acetylation in *M. marinum*. We renamed MMAR_1839, ESX-1 modifying protein-1 (Emp1).

To determine if Emp1 was required for the acetylation of additional mycobacterial proteins, we performed LFQ mass spectrometry to measure the relative changes in acetylation and protein levels in the Δ*emp1* strain as compared to the WT and complemented strains (Δ*emp1/*comp, Data set S1, Raw and Trimmed Data tabs S1A and S2B; the raw data have been uploaded to MassIVE due to size requirements and can be found at ftp://MSV000091442@massive.ucsd.edu). EsxA levels were comparable in all three strains (Fig. 2C; gray bars, Data set S1; Table S1C). Ac-EsxA was detected in the WT and the complemented strains but not in the Δ*emp1* strain. From these data, we conclude that Emp1 is the only NAT responsible for EsxA acetylation in *M. marinum*.

We next performed *k*-means (62) clustering analysis for each N-terminally acetylated protein in the data set, using the biological replicate with the best coverage (Data set S1; Table S1C). This approach systematically identified patterns between the WT, ∆*emp1*, and complementation strains across every protein. The variables considered for the clustering were from the following LFQ area ratios: Δ*emp1*:Δ*emp1/*comp, Δ*emp1*:WT, and Δ*emp1*/comp:WT. The proteins were clustered into three groups, using 25 random starting points. We reasoned that proteins that clustered with EsxA were potential acetylation targets of Emp1, as they also exhibited loss of acetylated intensity in the ∆*emp1* strain, which was restored upon complementation. The acetylation intensity patterns of proteins identified from the clustering were compared across all biological replicates.

This approach revealed a cluster of 22 proteins whose N-terminal acetylation followed a similar pattern to Ac-EsxA. All 23 proteins, including EsxA, exhibited undetectable levels of N-terminal acetylation in the Δ*emp1* strain, and restoration of acetylation in the Δ*emp1*/comp strain, similar to the WT strain (Fig. 2E; EsxA highlighted in pink, Data set Table S1E). Functional analysis using annotations in Mycobrowser (63) revealed that proteins dependent on Emp1 for acetylation are predicted to function in lipid metabolism or intermediary metabolism and respiration, and virulence (Fig. 2E). More than half are annotated as essential *in vitro* in *M. tuberculosis*. Finally, comparing the N-terminal sequences of the putative Emp-1 targets against the entire *M. marinum* proteome revealed a strong negative bias for basic residues within these first 10 amino acids (Fig. 2F, upper), likely due to the use of trypsin for the mass-spectrometry-based proteomics, which cleaves after Lys and Arg. Consequently, those peptides are underrepresented in the first 10 amino acids of the N-termini because they would not be observed due to their small size (43, 54). There was also a strong preference for threonine, with a mild preference for serine at the second amino acid position, consistent with our prior work (43). Comparison of the N-terminal sequences of the putative Emp1-targets against the *M. marinum* N-terminal acetylome (43) revealed a strong preference for threonine and glutamic acid at the second and third amino acid positions and a significant underrepresentation of alanine at the sixth amino acid position (Fig. 2F, lower). Together, these data demonstrate that Emp1 is required for the N-terminal acetylation of EsxA and at least 22 other proteins in *M. marinum*.

### Emp1, and therefore EsxA acetylation, is dispensable for EsxA/EsxB secretion from *M. marinum*

**Fig 3:**
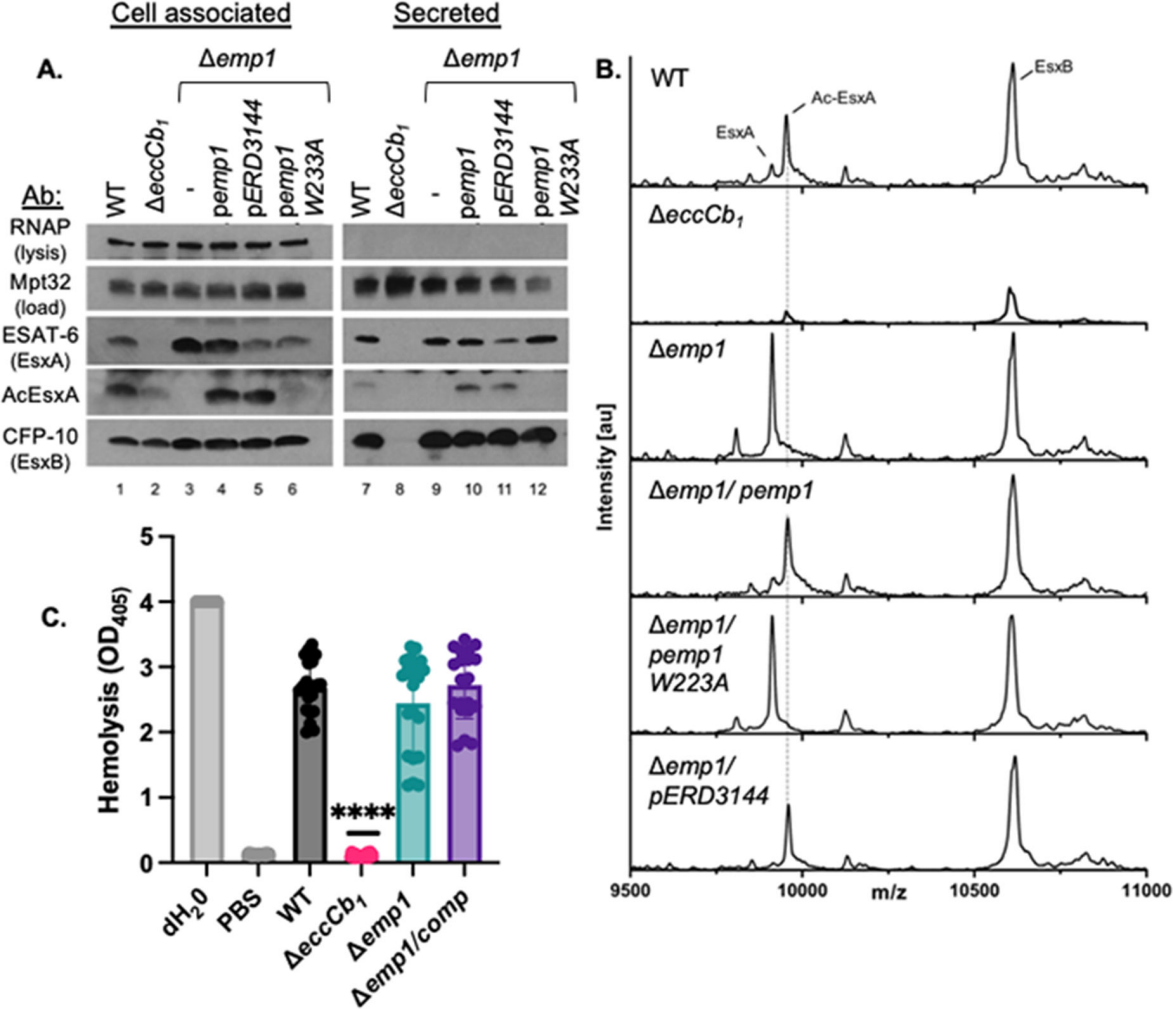
Emp1 is dispensable for EsxA and EsxB stability and secretion from *M. marinum*. (A) Western blot analysis of cell-associated and secreted proteins from *M. marinum* in the presence and absence of Emp1. (B) Whole colony MALDI-TOF MS. Spectra generated by whole colony MALDI-TOF for *M. marinum* strains are shown. The labeled peaks correspond to EsxA (9,915 m/z), Ac-EsxA (9,957 m/z), and EsxB(10,606 m/z Da), respectively. The dotted line was added for clarity. (C) Hemolytic activity of *M. marinum* strains. The data shown include seven biological replicates each in technical triplicate. Each data point is a technical replicate. Statistical analysis was performed using an ordinary one-way analysis of variance (ANOVA) (*P* < 0.0001) followed by a Tukey’s multiple comparison test. The significance shown is compared to the WT strain, ****P < 0.0001. Other important comparisons are discussed in the text.

The identification of Emp1 allowed us to test the role of EsxA acetylation on ESX-1 function in the presence of the WT *esxA* gene in *M. marinum*. It was previously suggested that EsxA acetylation impacted the interaction between EsxA and its binding partner, EsxB (30). The EsxA-EsxB interaction is required for ESX-1 function because it is required for protein stability and for targeting the EsxA-B pair to the ESX-1 system (6, 56, 64). If N-terminal acetylation of EsxA was required for EsxA-EsxB interaction then we would expect a loss of EsxB protein from the Δ*emp1* strain. We generated cell-associated and secreted protein fractions from *M. marinum* strains. EsxA, Ac-EsxA, and EsxB were produced (Fig. 3A, lane 1) and secreted (lane 7) from the WT *M. marinum* strain. EsxA and Ac-EsxA levels were reduced in the Δ*eccCb_1_* strain (lane 2), consistent with the reduced levels of EsxA in the absence of secretion (6, 65). Neither EsxA, Ac-EsxA, nor EsxB was secreted from the Δ*eccCb_1_* strain (lane 8). Although EsxA and EsxB were produced in the Δ*emp1* strain, Ac-EsxA was not detected (lane 3). EsxA and EsxB were secreted from the Δ*emp1* strain (lane 9). Constitutive expression of the *emp1* gene restored the production (lane 4) and secretion of Ac-EsxA (lane 10). Likewise, expression of the orthologous gene from *M. tuberculosis* Erdman (*ERD_3144*) restored the production and secretion of Ac-EsxA from the Δ*emp1* strain (lanes 5 and 11). Finally, we mutated the predicted active site of Emp1 (W223A), which would render the enzyme unable to bind Ac-CoA (66). Expression of *emp1W223A* in the Δ*emp1* strain did not restore the production or secretion of Ac-EsxA (lanes 6 and 12). Together, these data demonstrate that Emp1 is required for the acetylation of EsxA. Contrary to the existing models (31), the loss of EsxA acetylation did not result in a loss of the EsxA or EsxB protein, suggesting that the acetylation state of EsxA is dispensable for EsxA/EsxB interaction and secretion from *M. marinum* during *in vitro* growth. Our data support that Emp1 likely functions as a NAT in *M. marinum* and is functionally conserved in *M. tuberculosis*.

We sought a more sensitive approach to verify that EsxA was completely unacetylated in the Δ*emp1* strain and rule out cross-talk by other putative NATs in the absence of *emp1*. We previously demonstrated that both Ac-EsxA and EsxA are resolved in proteins washed from the surface of *M. marinum* colonies using whole-colony MALDI-TOF-MS (58). Using this approach, we detected peaks consistent with both unacetylated EsxA (9,915 m/z) and Ac-EsxA (9,957 m/z) from surface-associated proteins isolated from WT *M. marinum* colonies (Fig. 3B). We also detected surface-associated EsxB (10,606 m/z). The Δ*eccCb_1_* strain is a lysis control because this strain produces but does not secrete EsxA and EsxB (6, 17). Both EsxA species and EsxB were significantly diminished from the proteins isolated from the surface of the Δ*eccCb_1_* strain (6, 58). Therefore, the observed peaks were due to the secretion of EsxA and EsxB to the cell surface. Proteins isolated from the surface of the Δ*emp1* strain resulted in a single EsxA peak which corresponded to the unacetylated EsxA protein and a peak for EsxB. The Ac-EsxA peak was completely abrogated. Expression of the WT *emp1* gene, but not the *emp1W223A* gene, restored the peak corresponding to the Ac-EsxA protein. From these data, we conclude that deletion of the *emp1* gene results in a complete loss of Ac-EsxA in *M. marinum*, demonstrating that Emp1 is solely responsible for the acetylation of EsxA *in vivo*. The absence of acetylation in the W223A active-site mutant of Emp-1 (Fig. 3B) demonstrates that functional Emp-1 is required for the acetylation of EsxA. Moreover, our findings show that EsxA and EsxB are secreted from *M. marinum* independently of EsxA-N-terminal acetylation during *in vitro* growth.

*M. marinum* lyses red blood cells in a contact-dependent, ESX-1-dependent manner (17). EsxA is required for the hemolytic activity of *M. marinum*, likely because it is required for the secretion of the majority of the ESX-1 substrates (5, 7, 33, 67). *M. marinum* strains that secrete all of the known ESX-1 substrates, including the late substrates, EspE and EspF, are hemolytic (17, 68). The Δ*esxA M. marinum* strain is non-hemolytic (5). Because the acetylation of EsxA depends on Emp1, we reasoned that if EsxA acetylation was required for EsxA-mediated secretion, the Δ*emp1* strain would have altered hemolytic activity. WT *M. marinum* lysed sheep red blood cells (sRBCs), while the Δ*eccCb_1_* strain (which fails to secrete ESX-1 substrates) exhibited significantly reduced hemolytic activity (Fig. 3C, *P* < 0.0001, relative to the WT strain). Water and phosphate-buffered saline (PBS) (cell-free) were used as positive and negative controls for hemolysis, respectively. The activity of the Δ*eccCb_1_* strain was not significantly different from the PBS control (*P* < 0.9999). The hemolytic activities of the Δ*emp1* and the Δ*emp1* complemented strains were not significantly different from the WT strain (*P* = 0.4837 and *P* = 0.9998) or each other (*P* = 0.2689). From these data, we conclude that Emp1 is dispensable for the hemolytic activity of *M. marinum*. Because ESX-1 mediates hemolysis, the data suggest that the acetylation of EsxA is also dispensable for hemolysis and is consistent with the secretion of EsxA and EsxB from the Δ*emp1* strain (Fig. 3B). Finally, because additional ESX-1 substrates required for hemolysis depend upon EsxA for secretion, our data suggest that the secretion of additional ESX-1 substrates occurs independently of EsxA acetylation.

### Emp1 is required for macrophage cytolysis and cell-to-cell spread of *M. marinum*

**Fig 4:**
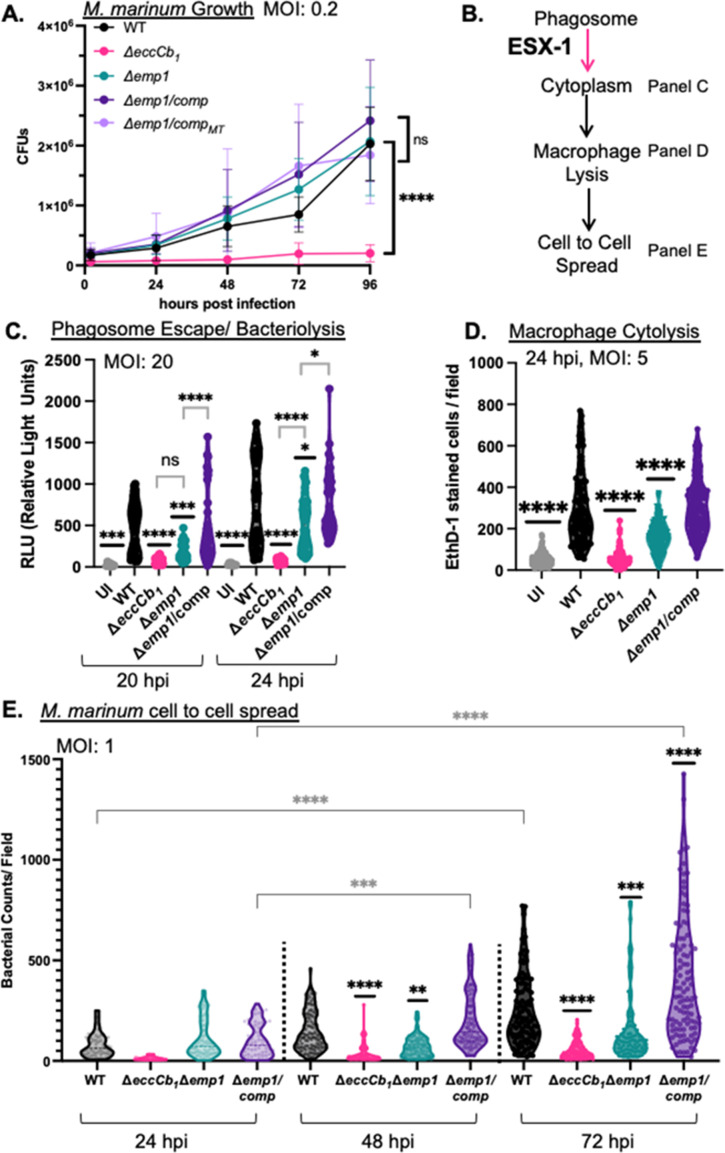
Emp1 is required for cytolytic activity and cell-to-cell spread of *M. marinum*. (A) CFU analysis of *M. marinum* strains. MOI = 0.2 plated in triplicate, represents three biological replicates. Significance was determined using a two-way RM ANOVA (*P* < 0.0001) followed by a Tukey’s multiple comparison test. The significance shown is compared to the WT strain, for the 96 hpi. However, the CFUs from the Δ*eccCb_1_* strain were significantly lower than the WT strain throughout the experiment. *P* values comparing Δ*eccCb_1_* vs WT were as follows: 0 hpi **P = 0.0035, 24 hpi, *P = 0.0162, 48 hpi ***P = 0.0001, and 72 hpi and 96 hpi ****P < 0.0001. The WT strain was not significantly different from the additional strains at any time point. (B) Schematic of progression through macrophage infection. “Panels” refer to this figure. (C) Cytoplasmic bacteriolysis of *M. marinum* strains in iIFNAR^−/−^ immortalized murine bone marrow-derived macrophages, at 20 and 24 hpi, MOI = 20. The data includes four biological replicates, each performed in three technical replicates. Outliers were identified and removed using ROUT analysis (Q = 0.5%). Cleaned data were tested for significance using a one-way ordinary ANOVA (*P <* 0.0001) followed by a Tukey’s multiple comparison test. *P = 0.0360, ***P = 0.0002, and ****P < 0.0001. (D) Cytolytic activity of *M. marinum* strains in RAW 264.7 cells, 24 hpi, MOI = 5. The data include at least three biological replicates each in technical triplicate, with 10 fields selected for each well. Each data point is the number of red cells per field. Significance was determined using an ordinary one-way ANOVA (*P* < 0.0001) followed by a Sidak’s multiple comparison test. The significance shown is compared to the WT strain, with additional comparisons discussed in the text. ****P < 0.0001. (E) Spread of *M. marinum* strains in a RAW 264.7 monolayer, at 24, 48, and 72 hpi, MOI = 1. The data include four biological replicates, each in technical triplicate, with 10 fields taken from each well, with the exception of the 24-hour timepoint. Each data point is the bacterial counts per field as measured by counting GFP “particles” using ImageJ. Significance was determined using an ordinary one-way ANOVA followed by a Tukey’s multiple comparison test. ****P < 0.0001. Black asterisks represent the comparison to the WT strain from the same timepoint. ***P = 0.0001 and **P = 0.0060. The gray asterisks represent the comparison between the 24 timepoint with the 48- or 72-hour timepoints for each strain. ***P = 0.0080. If no asterisks are listed, the *P* value is not significant.

It was previously reported that N-terminal acetylation of EsxA was required for ESX-1 function during infection (31). We reasoned that if acetylation of EsxA was required for ESX-1 function then the Δ*emp1 M. marinum* strain would phenocopy the Δ*eccCb_1_* strain during macrophage infection (6, 69, 70). We infected RAW 264.7 cells with *M. marinum* at a multiplicity of infection (MOI) of 0.2 and measured colony-forming units over time. The WT *M. marinum* strain grew in the macrophages, while the Δ*eccCb_1_* strain was attenuated for growth (Fig. 4A, *P* < 0.0001, relative to all of the additional strains). Growth of the Δ*emp1* and the Δ*emp1* complemented strains was not significantly different from the WT strain. We conclude that the Δ*emp1* strain is not attenuated for growth during macrophage infection, distinct from Δ*eccCb_1_* strain.

ESX-1-dependent phagosomal damage allows *M. marinum* translocation to the macrophage cytoplasm (Fig. 4B). It was previously reported that the N-terminal acetylation of EsxA was required for ESX-1-dependent phagosomal lysis (31). To test if the Δ*emp1* strain escapes the phagosome, we adopted a reporter used to reflect phagosomal escape and cytoplasmic bacteriolysis in *Listeria monocytogenes* and in *M. tuberculosis* (9, 71). Bacterial escape from the phagosome and subsequent bacteriolysis in the macrophage cytoplasm releases a reporter plasmid, allowing plasmid translocation to the nucleus and transcription of the *luc+* gene, causing luminescence. Phagosomal bacteria do not cause luminescence because the reporter plasmid does not access the cytoplasm. We adapted the reporter plasmid by adding an episomal mycobacterial origin and introduced it into *M. marinum.* We optimized the assay to distinguish between the cytoplasmic WT *M. marinum* strain and the phagosome-localized Δ*eccCb_1_* strain (*P* < 0.0001, Fig. S3B). Expression of the *eccCb_1_* gene in the Δ*eccCb_1_* strain significantly restored luminescence levels. These data confirm that the bacteriolysis reporter distinguishes between phagosomal and cytoplasmic *M. marinum* strains and measures cytoplasmic *M. marinum* bacteriolysis.

We infected iIFNAR^−/−^ macrophages at an MOI of 20 and measured luminescence at 20 and 24 hours post infection (Fig. 4C). We reasoned that if acetylation of EsxA was required for phagosomal escape then the Δ*emp1 M. marinum* strain would be retained in the phagosome, similar to the Δ*eccCb_1_* strain. At 20 hours post infection, the Δ*emp1* strain resulted in luminescence levels similar to the Δ*eccCb_1_* strain (*P* = 0.6191), significantly lower than the WT and complemented strain (*P* = 0.0003 vs WT, *P* = 0.0001 vs Δ*emp1*/comp). However, by 24 hours post infection, the Δ*emp1* strain resulted in significantly higher levels of luminescence than the Δ*eccCb_1_* strain (*P* < 0.0001), although still significantly lower than both the WT (*P* = 0.0264) and complemented (*P* = 0.0195) strains. From these data, we conclude that the Δ*emp1* strain is not retained in the phagosome like the Δ*eccCb_1_* strain, suggesting that the N-terminal acetylation of EsxA is not essential for phagosomal escape. Our data suggest that the Δ*emp1* strain is either released from the phagosome less efficiently than the WT strain or exhibits delayed or reduced bacteriolysis in the macrophage cytoplasm compared to the WT strain.

Release of *M. marinum* into the cytoplasm promotes macrophage cytolysis through ESX-1-independent mechanisms (Fig. 4B) (4, 11, 12, 72 – 75). To test if Emp1 was required for macrophage cytolysis, we infected RAW 264.7 cells with *M. marinum* at an MOI of 5 and measured uptake of the membrane impermeable dye, ethidium homodimer 1 (EthD-1). Infection with WT *M. marinum* resulted in significant cytolysis, as reflected by EthD-1 uptake, compared to the uninfected cells (Fig. 4D, *P* < 0.0001). The Δ*eccCb_1_* strain was significantly less cytolytic than the WT strain (*P* < 0.0001), similar to the uninfected control. The Δ*emp1* strain was significantly less cytolytic than the WT strain (*P* < 0.0001) but significantly more cytolytic than the Δ*eccCb_1_* strain (*P* < 0.0001). Constitutive expression of the *emp1* gene in the Δ*emp1* strain restored cytolysis to levels similar to the WT strain. Representative images of macrophage cytolysis are shown in Fig. S5. To confirm that these strains were not attenuated due to the spontaneous loss of the outer lipid phthiocerol dimycocerosates (PDIM), we performed thin-layer chromatography (TLC) analysis. All of the strains produced PDIM similar to the WT strain (Fig. S6). From these data, we conclude that Emp1 is required for macrophage cytolysis.

Macrophage cytolysis precedes cell-to-cell spread of *M. marinum* (Fig. 4A). We tested if Emp1 was required for *M. marinum* to spread between macrophages. To test the ability of *M. marinum* to spread, we introduced a plasmid that expresses green fluorescent protein (GFP) from a tet-ON promoter (the promoter is turned on by the addition of ATc) into the WT, Δ*eccCb_1_*, Δ*emp1*, and complemented strains. We measured GFP expression in the *M. marinum* cells grown under laboratory conditions to confirm that GFP was expressed in all strains (Fig. S7). We infected RAW 264.7 cells with *M. marinum* at an MOI of 1 and measured the spread of the GFP-labeled *M. marinum* cells across the macrophage monolayer. Following infection and treatment to kill the extracellular bacteria, we overlaid the monolayer with 0.8% agarose to prevent the movement of the macrophages. We observed the GFP signal using microscopy every 24 hours for 72 hours post infection. As shown in Fig. 4E, infection of RAW 264.7 cells with WT *M. marinum* resulted in detectable spread of *M. marinum* across the monolayer, as reflected by increasing GFP signal over time (black violin plots, *P* < 0.0001 between the 24 hour and 72 hour timepoints). The Δ*eccCb_1_* strain exhibited significantly less spread than the WT strain at 48 and 72 hours post infection (*P* < 0.0001), and there was no significant difference between the GFP signals generated from the Δ*eccCb_1_* infections over the course of the experiment (pink violin plots). The lack of spread of the Δ*eccCb_1_* strain was consistent with phagosomal retention, attenuated growth during infection and reduced macrophage cytolysis (Fig. 4B through D). Interestingly, although the Δ*emp1* strain grew to WT levels during infection (Fig. 4A), it exhibited significantly less spread than the WT strain at 48 and 72 hours post infection (*P* = 0.0060 and *P* = 0.0001), with no significant difference between the GFP signals generated over the course of the experiment. These data are consistent with the reduced macrophage cytolysis measured in Fig. 4D. Expression of *emp1* in the Δ*emp1* strain (Δ*emp1/comp*) restored cell-to-cell spread (purple violin plots, *P* < 0.0001 at 72 hours relative to the 24 hours timepoint, *P* = 0.0080 at 48 hours relative to the 24 hours timepoint) to levels significantly higher than the WT strain at 72 hpi (*P* < 0.0001). Representative images are shown in Fig. S8. These data support that *emp1* is required for spread of *M. marinum* between macrophages. Overall, our data support that the attenuation of the Δ*emp1* strain occurs downstream of ESX-1 function.

## DISCUSSION

In this study, we demonstrated that Emp1, a predicted NAT, is required for the N-terminal acetylation of EsxA and other mycobacterial proteins. We demonstrated functional conservation between the *M. tuberculosis* and *M. marinum emp1* genes. We found that Emp1 is solely responsible for the acetylation of EsxA and other mycobacterial proteins. We demonstrated that Emp1 is dispensable for ESX-1-dependent secretion and hemolysis and for growth in macrophages during infection. However, Emp1 was required for WT levels of cytoplasmic translocation or survival, macrophage cytolysis, and cell-to-cell spread of *M. marinum.* Collectively, this study identified a NAT required for N-terminal acetylation in *Mycobacterium* and provided insight into the requirement of N-terminal acetylation of EsxA and other proteins for mycobacterial virulence in the macrophage.

Although ~10%–15% of the mycobacterial proteome is likely N-terminally acetylated (43), little is known about the NAT enzymes responsible for N-terminal acetylation in *Mycobacterium.* Prior studies aimed at understanding N-terminal acetylation have focused on EsxA. The initial study demonstrating N-terminal acetylation of EsxA suggested that EsxA acetylation impacted the interaction with its binding partner, EsxB (30). If this were the case, we would have expected a loss of EsxA and EsxB protein in the Δ*emp1* strain, similar to the Δ*esxA* strain. Instead, EsxA and EsxB were made and secreted from *M. marinum* in the Δ*emp1* strain. Aguilera et al. mutated the Thr residue at the second position of EsxA, reporting a loss of cytoplasmic translocation by FRET assay and reduced macrophage cytolysis (31). They found no significant difference in the FRET assay between the phagosome associated Δ*esxA* strain and the strains with unacetylated EsxA variants, leading to the conclusion that N-terminal acetylation of EsxA was required for phagosomal lysis and macrophage cytolysis (31). In agreement with this study, abrogation of EsxA acetylation through the deletion of *emp1* resulted in a significant reduction of macrophage cytolysis. However, we do not attribute the reduced cytolysis to a loss of ESX-1 function for several reasons. First, our prior work demonstrates that EsxA is required for ESX-1-dependent secretion and hemolytic activity because it is required for the secretion of ESX-1 substrates (5, 67). We have reported attenuated and non-hemolytic *M. marinum* strains that secrete WT levels of EsxA and EsxB (5, 67, 69). If N-terminal acetylation was essential for EsxA function, we would have expected a loss of both protein secretion from and hemolytic activity of the Δ*emp1* strain. Instead, neither secretion nor hemolysis were dependent on Emp1. Second, if unacetylated EsxA was nonfunctional, we would have expected the Δ*emp1* strain to phenocopy the Δ*eccCb_1_* strain during macrophage infection. Instead, Δ*emp1* strain grew similarly in macrophages to the WT strain, and the Δ*emp1* strain accessed the cytoplasm. Our findings are consistent with the Δ*emp1* strain undergoing reduced bacteriolysis. Decreased bacteriolysis would cause less bacterial DNA in the cytoplasm, reducing pyroptosis, which is a cause of cytolysis (9, 71, 76), consistent with our other data. We suspect changing the second residue of EsxA to modulate acetylation abrogated EsxA function, causing a loss of secretion, a lack of phagosomal escape, and reduced cytolysis. In our study, the unacetylated EsxA protein retains its WT sequence and clearly promotes ESX-1-dependent secretion and virulence, supporting that N-terminal acetylation is dispensable for ESX-1 function under the conditions tested in this study, moving the field of type VII secretion forward. It remains unclear why EsxA is N-terminally acetylated, but it is formally possible that EsxA N-terminal acetylation may contribute to cytolysis downstream of phagosomal lysis.

We propose that the Emp1-dependent N-terminal acetylation of another protein or proteins is required for macrophage cytolysis, downstream of ESX-1 function. For example, Emp-1 dependent N-terminal acetylation may stabilize select mycobacterial proteins, consistent with N-terminal acetylation in higher organisms (25, 44). Alternatively, a specific Emp1 target may promote macrophage cytolysis and cell-to-cell spread. Regardless of the mechanism, Emp1 is a bacterial enzyme specifically required for macrophage cytolysis and cell-to-cell spread but not for the growth of the pathogen. Therapeutics that target Emp1 activity may be a new way to stop mycobacterial infections by limiting cell-to-cell spread of mycobacterial pathogens.

In general, *M. marinum* strains that are hemolytic are also cytolytic. The Δ*emp1* strain is hemolytic but not cytolytic. We previously published an *M. marinum* strain that was non-hemolytic but retained cytolytic activity, demonstrating the two activities can be separable. One reason for the disconnect between growth and cytolysis could be due to the permissive nature of RAW cells for mycobacterial growth, and this may not reflect growth in other macrophage types.

Here, we advance the field of mycobacterial physiology by identifying a NAT that promotes N-terminal acetylation, contributing to the basic understanding of this fundamental protein modification in bacteria. The framework we provide is widely accessible and applicable to any system and will facilitate the identification of NATs required for the N-terminal acetylation of specific protein targets in any organism. Importantly, we showed that an antibody against an acetylated N-terminal peptide could discriminate between acetylated and unacetylated N-termini. The generation of similar antibodies for additional N-terminally acetylated proteins could be used in any system to demonstrate N-terminal acetylation and identify the responsible NAT. We will continue to address which of the additional putative NATs contribute to N-terminal acetylation in *Mycobacterium* as well as their breadth of function and specificity. Furthermore, because Emp1 is required for a subset of the N-terminally acetylated mycobacterial proteins, we will focus on understanding the specificity of Emp1.

One limitation of this study is that we were unable to show the Emp1 was sufficient and necessary for the acetylation of EsxA *in vitro*. This would support the hypothesis that Emp1 directly acetylates EsxA at its N-terminus. We expressed the Emp1 and the Emp1W223A versions in *E. coli* with the goal of purification from a heterologous host. Despite trying different temperatures, additives, and vectors, we were unable to generate and isolate soluble forms of the proteins. Instead, we expressed Emp1 in *E. coli* and incubated the resulting lysate to acetylate a series of EsxA N-terminal peptides. Finally, we tried co-expressing *emp1* and either *esxA* or *esxBA* in *E. coli* and measuring EsxA acetylation using western blot analysis. We were unable to observe acetylation using these approaches. We are uncertain why we are unable to produce functional Emp1 protein *in vitro* or in *E. coli*, while we can express and purify functional NATs from *E.coli* and *Salmonella typhimurium* (RimI). We suspect that Emp1 requires additional, unidentified cofactors or environmental cues for function that are specific to *Mycobacterium.*

Overall, this study contributes a fundamental understanding of the conserved mechanisms and underlying N-terminal protein acetylation in pathogenic mycobacteria and identifies the NAT solely responsible for EsxA acetylation in *M. marinum*, opening new avenues of study aimed at further understanding this protein modification and its function in bacteria.

## MATERIALS AND METHODS

*M. marinum* strains were grown as described previously (5). Plasmids were constructed using FAST Cloning or restriction cloning and maintained in *E. coli* as described (5, 69, 77). *M. marinum* strains were constructed using allelic exchange (5, 69, 77). N-terminal acetylation was measured using dot blot and NUT-PAGE followed by western blot analysis. Protein production and secretion were measured using western blot analysis as previously described (5). Site directed mutagenesis of the *emp1* gene was performed as in references (56, 57). Whole colony MALDI mass spectrometry to measure surface-associated EsxA, Ac-EsxA, and EsxB was performed as in reference (58). LFQ mass spectrometry was used to measure N-terminal acetylation from *M. marinum* whole cell lysates, similar to references (70, 78). Hemolytic activity of *M. marinum* was measured against sRBCs as described (5). Thin-layer chromatography was used to confirm PDIM production in the Δ*emp1* strain (79). iIFNAR^−/−^ macrophages were used with a luciferase reporter to indirectly measure release of *M. marinum* from the phagosome and survival in the macrophage cytoplasm. RAW264.7 cells were used as an infection model to measure growth of *M. marinum* during infection and macrophage cytolysis as described previously (5). Spread of *M. marinum* across the RAW264.7 monolayer was analyzed based on these studies (16, 17). Bioinformatic analysis and statistical analysis were performed using Prism and R Studio. Detailed Materials and Methods are available in the Supplementary Material.

## SUPPLEMENTAL MATERIAL

Supplemental Material mbio.00987-23-s0001.pdf Figures S1–S8, Tables S1 and S2, supplemental methods, data set caption, and supplemental references.

Supplemental Data Set mbio.00987-23-s0002.xlsx Proteomics data.

ASM does not own the copyrights to Supplemental Material that may be linked to, or accessed through, an article. The authors have granted ASM a non-exclusive, world-wide license to publish the Supplemental Material files. Please contact the corresponding author directly for reuse.

## References

[1] Sørensen AL , Nagai S , Houen G , Andersen P , Andersen AB . 1995. Purification and characterization of a low-molecular-mass T-cell antigen secreted by Mycobacterium tuberculosis. Infect Immun 63:1710–1717. doi:10.1128/iai.63.5.1710-1717.1995 7729876 PMC173214

[2] Andersen P , Andersen AB , Sørensen AL , Nagai S . 1995. Recall of long-lived immunity to Mycobacterium tuberculosis infection in mice. J Immunol 154:3359–3372. 7897219

[3] Berthet F-X , Rasmussen PB , Rosenkrands I , Andersen P , Gicquel B . 1998. A Mycobacterium tuberculosis operon encoding ESAT-6 and a novel low-molecular-mass culture filtrate protein (CFP-10). Microbiology (Reading) 144 (Pt 11):3195–3203. doi:10.1099/00221287-144-11-3195 9846755

[4] Hsu T , Hingley-Wilson SM , Chen B , Chen M , Dai AZ , Morin PM , Marks CB , Padiyar J , Goulding C , Gingery M , Eisenberg D , Russell RG , Derrick SC , Collins FM , Morris SL , King CH , Jacobs WR Jr . 2003. The primary mechanism of attenuation of bacillus Calmette-Guerin is a loss of secreted lytic function required for invasion of lung interstitial tissue. Proc Natl Acad Sci U S A 100:12420–12425. doi:10.1073/pnas.1635213100 14557547 PMC218773

[5] Cronin RM , Ferrell MJ , Cahir CW , Champion MM , Champion PA . 2022. Proteo-genetic analysis reveals clear hierarchy of ESX-1 secretion in Mycobacterium marinum. Proc Natl Acad Sci U S A 119:e2123100119. doi:10.1073/pnas.2123100119 35671426 PMC9214503

[6] Stanley SA , Raghavan S , Hwang WW , Cox JS . 2003. Acute infection and macrophage subversion by Mycobacterium tuberculosis require a specialized secretion system. Proc Natl Acad Sci U S A 100:13001–13006. doi:10.1073/pnas.2235593100 14557536 PMC240734

[7] Fortune SM , Jaeger A , Sarracino DA , Chase MR , Sassetti CM , Sherman DR , Bloom BR , Rubin EJ . 2005. Mutually dependent secretion of proteins required for mycobacterial virulence. Proc Natl Acad Sci U S A 102:10676–10681. doi:10.1073/pnas.0504922102 16030141 PMC1176248

[8] Guinn KM , Hickey MJ , Mathur SK , Zakel KL , Grotzke JE , Lewinsohn DM , Smith S , Sherman DR . 2004. Individual RD1-region genes are required for export of ESAT-6/CFP-10 and for virulence of Mycobacterium tuberculosis. Mol Microbiol 51:359–370. doi:10.1046/j.1365-2958.2003.03844.x 14756778 PMC1458497

[9] Manzanillo PS , Shiloh MU , Portnoy DA , Cox JS . 2012. Mycobacterium tuberculosis activates the DNA-dependent cytosolic surveillance pathway within macrophages. Cell Host Microbe 11:469–480. doi:10.1016/j.chom.2012.03.007 22607800 PMC3662372

[10] Conrad WH , Osman MM , Shanahan JK , Chu F , Takaki KK , Cameron J , Hopkinson-Woolley D , Brosch R , Ramakrishnan L . 2017. Mycobacterial ESX-1 secretion system mediates host cell lysis through bacterium contact-dependent gross membrane disruptions. Proc Natl Acad Sci U S A 114:1371–1376. doi:10.1073/pnas.1620133114 28119503 PMC5307465

[11] Houben D , Demangel C , van Ingen J , Perez J , Baldeón L , Abdallah AM , Caleechurn L , Bottai D , van Zon M , de Punder K , van der Laan T , Kant A , Bossers-de Vries R , Willemsen P , Bitter W , van Soolingen D , Brosch R , van der Wel N , Peters PJ . 2012. ESX-1-mediated translocation to the cytosol controls virulence of mycobacteria. Cell Microbiol 14:1287–1298. doi:10.1111/j.1462-5822.2012.01799.x 22524898

[12] van der Wel N , Hava D , Houben D , Fluitsma D , van Zon M , Pierson J , Brenner M , Peters PJ . 2007. M. tuberculosis and M. leprae translocate from the phagolysosome to the cytosol in myeloid cells. Cell 129:1287–1298. doi:10.1016/j.cell.2007.05.059 17604718

[13] Mehra A , Zahra A , Thompson V , Sirisaengtaksin N , Wells A , Porto M , Köster S , Penberthy K , Kubota Y , Dricot A , Rogan D , Vidal M , Hill DE , Bean AJ , Philips JA . 2013. Mycobacterium tuberculosis type VII secreted effector EsxH targets host ESCRT to impair trafficking. PLoS Pathog 9:e1003734. doi:10.1371/journal.ppat.1003734 24204276 PMC3814348

[14] Simeone R , Bobard A , Lippmann J , Bitter W , Majlessi L , Brosch R , Enninga J . 2012. Phagosomal rupture by Mycobacterium tuberculosis results in toxicity and host cell death. PLoS Pathog 8:e1002507. doi:10.1371/journal.ppat.1002507 22319448 PMC3271072

[15] Watson RO , Manzanillo PS , Cox JS . 2012. Extracellular M. tuberculosis DNA targets bacteria for autophagy by activating the host DNA-sensing pathway. Cell 150:803–815. doi:10.1016/j.cell.2012.06.040 22901810 PMC3708656

[16] Castro-Garza J , King CH , Swords WE , Quinn FD . 2002. Demonstration of spread by Mycobacterium tuberculosis bacilli in A549 epithelial cell monolayers. FEMS Microbiol Lett 212:145–149. doi:10.1111/j.1574-6968.2002.tb11258.x 12113926

[17] Gao L-Y , Guo S , McLaughlin B , Morisaki H , Engel JN , Brown EJ . 2004. A mycobacterial virulence gene cluster extending RD1 is required for cytolysis, bacterial spreading and ESAT-6 secretion. Mol Microbiol 53:1677–1693. doi:10.1111/j.1365-2958.2004.04261.x 15341647

[18] Derrick SC , Morris SL . 2007. The ESAT6 protein of Mycobacterium tuberculosis induces apoptosis of macrophages by activating caspase expression. Cell Microbiol 9:1547–1555. doi:10.1111/j.1462-5822.2007.00892.x 17298391

[19] Lin J , Chang Q , Dai X , Liu D , Jiang Y , Dai Y . 2019. Early secreted antigenic target of 6-kDa of Mycobacterium tuberculosis promotes caspase-9/caspase-3-mediated apoptosis in macrophages. Mol Cell Biochem 457:179–189. doi:10.1007/s11010-019-03522-x 30911956

[20] Grover A , Izzo AA . 2012. BAT3 regulates Mycobacterium tuberculosis protein ESAT-6-mediated apoptosis of macrophages. PLoS One 7:e40836. doi:10.1371/journal.pone.0040836 22808273 PMC3396635

[21] Pagán AJ , Lee LJ , Edwards-Hicks J , Moens CB , Tobin DM , Busch-Nentwich EM , Pearce EL , Ramakrishnan L . 2022. MTOR-regulated mitochondrial metabolism limits Mycobacterium-induced cytotoxicity. Cell 185:3720–3738. doi:10.1016/j.cell.2022.08.018 36103894 PMC9596383

[22] Osman MM , Shanahan JK , Chu F , Takaki KK , Pinckert ML , Pagán AJ , Brosch R , Conrad WH , Ramakrishnan L . 2022. The C terminus of the Mycobacterium ESX-1 secretion system substrate ESAT-6 is required for phagosomal membrane damage and virulence. Proc Natl Acad Sci U S A 119:e2122161119. doi:10.1073/pnas.2122161119 35271388 PMC8931374

[23] Choi H-H , Shin D-M , Kang G , Kim K-H , Park JB , Hur GM , Lee H-M , Lim Y-J , Park J-K , Jo E-K , Song C-H . 2010. Endoplasmic reticulum stress response is involved in Mycobacterium tuberculosis protein ESAT-6-mediated apoptosis. FEBS Lett 584:2445–2454. doi:10.1016/j.febslet.2010.04.050 20416295

[24] Romagnoli A , Etna MP , Giacomini E , Pardini M , Remoli ME , Corazzari M , Falasca L , Goletti D , Gafa V , Simeone R , Delogu G , Piacentini M , Brosch R , Fimia GM , Coccia EM . 2012. ESX-1 dependent impairment of autophagic flux by Mycobacterium tuberculosis in human dendritic cells. Autophagy 8:1357–1370. doi:10.4161/auto.20881 22885411 PMC3442882

[25] Aksnes H , Ree R , Arnesen T . 2019. Post-translational, and non-catalytic roles of N-terminal acetyltransferases. Molecular Cell 73:1097–1114. doi:10.1016/j.molcel.2019.02.007 30878283 PMC6962057

[26] Favrot L , Blanchard JS , Vergnolle O . 2016. Bacterial GCN5-related N-acetyltransferases: from resistance to regulation. Biochemistry 55:989–1002. doi:10.1021/acs.biochem.5b01269 26818562 PMC4795176

[27] Polevoda B , Sherman F . 2003. N-terminal acetyltransferases and sequence requirements for N-terminal acetylation of eukaryotic proteins. J Mol Biol 325:595–622. doi:10.1016/s0022-2836(02)01269-x 12507466

[28] Arfin SM , Kendall RL , Hall L , Weaver LH , Stewart AE , Matthews BW , Bradshaw RA . 1995. Eukaryotic methionyl aminopeptidases: two classes of cobalt-dependent enzymes. Proc Natl Acad Sci U S A 92:7714–7718. doi:10.1073/pnas.92.17.7714 7644482 PMC41216

[29] Driessen HP , de Jong WW , Tesser GI , Bloemendal H . 1985. The mechanism of N-terminal acetylation of proteins. CRC Crit Rev Biochem 18:281–325. doi:10.3109/10409238509086784 3902358

[30] Okkels LM , Müller E-C , Schmid M , Rosenkrands I , Kaufmann SHE , Andersen P , Jungblut PR . 2004. CFP10 discriminates between nonacetylated and acetylated ESAT-6 of Mycobacterium tuberculosis by differential interaction. Proteomics 4:2954–2960. doi:10.1002/pmic.200400906 15378760

[31] Aguilera J , Karki CB , Li L , Vazquez Reyes S , Estevao I , Grajeda BI , Zhang Q , Arico CD , Ouellet H , Sun J . 2020. N (alpha)-acetylation of the virulence factor EsxA is required for mycobacterial cytosolic translocation and virulence. J Biol Chem 295:5785–5794. doi:10.1074/jbc.RA119.012497 32169899 PMC7186180

[32] Tobin DM , Ramakrishnan L . 2008. Comparative pathogenesis of Mycobacterium marinum and Mycobacterium tuberculosis. Cell Microbiol 10:1027–1039. doi:10.1111/j.1462-5822.2008.01133.x 18298637

[33] McLaughlin B , Chon JS , MacGurn JA , Carlsson F , Cheng TL , Cox JS , Brown EJ . 2007. A Mycobacterium ESX-1-secreted virulence factor with unique requirements for export. PLoS Pathog 3:e105. doi:10.1371/journal.ppat.0030105 17676952 PMC1937011

[34] Aksnes H , Marie M , Arnesen T , Drazic A . 2018. Actin polymerization and cell motility are affected by NAA80-mediated posttranslational N-terminal acetylation of actin. Commun Integr Biol 11:e1526572. doi:10.1080/19420889.2018.1526572 30534344 PMC6284563

[35] Drazic A , Aksnes H , Marie M , Boczkowska M , Varland S , Timmerman E , Foyn H , Glomnes N , Rebowski G , Impens F , Gevaert K , Dominguez R , Arnesen T . 2018. NAA80 is actin’s N-terminal acetyltransferase and regulates cytoskeleton assembly and cell motility. Proc Natl Acad Sci U S A 115:4399–4404. doi:10.1073/pnas.1718336115 29581253 PMC5924898

[36] Linster E , Wirtz M . 2018. N-terminal acetylation: an essential protein modification emerges as an important regulator of stress responses. J Exp Bot 69:4555–4568. doi:10.1093/jxb/ery241 29945174

[37] Aksnes H , Van Damme P , Goris M , Starheim KK , Marie M , Støve SI , Hoel C , Kalvik TV , Hole K , Glomnes N , Furnes C , Ljostveit S , Ziegler M , Niere M , Gevaert K , Arnesen T . 2015. An organellar nalpha-acetyltransferase, NAA60, acetylates cytosolic N termini of transmembrane proteins and maintains Golgi integrity. Cell Rep 10:1362–1374. doi:10.1016/j.celrep.2015.01.053 25732826

[38] Dikiy I , Eliezer D . 2014. N-terminal acetylation stabilizes N-terminal helicity in lipid- and micelle-bound alpha-synuclein and increases its affinity for physiological membranes. J Biol Chem 289:3652–3665. doi:10.1074/jbc.M113.512459 24338013 PMC3916564

[39] Shemorry A , Hwang CS , Varshavsky A . 2013. Control of protein quality and stoichiometries by N-terminal acetylation and the N-end rule pathway. Mol Cell 50:540–551. doi:10.1016/j.molcel.2013.03.018 23603116 PMC3665649

[40] Van Damme P , Hole K , Pimenta-Marques A , Helsens K , Vandekerckhove J , Martinho RG , Gevaert K , Arnesen T , Snyder M . 2011. NatF contributes to an evolutionary shift in protein N-terminal acetylation and is important for normal chromosome segregation. PLoS Genet 7:e1002169. doi:10.1371/journal.pgen.1002169 21750686 PMC3131286

[41] Hwang CS , Shemorry A , Varshavsky A . 2010. N-terminal acetylation of cellular proteins creates specific degradation signals. Science 327:973–977. doi:10.1126/science.1183147 20110468 PMC4259118

[42] Behnia R , Panic B , Whyte JRC , Munro S . 2004. Targeting of the Arf-like GTPase Arl3P to the Golgi requires N-terminal acetylation and the membrane protein Sys1p. Nat Cell Biol 6:405–413. doi:10.1038/ncb1120 15077113

[43] Thompson CR , Champion MM , Champion PA . 2018. Quantitative N-terminal footprinting of pathogenic mycobacteria reveals differential protein acetylation. J Proteome Res 17:3246–3258. doi:10.1021/acs.jproteome.8b00373 30080413 PMC6264890

[44] Christensen DG , Baumgartner JT , Xie X , Jew KM , Basisty N , Schilling B , Kuhn ML , Wolfe AJ . 2019. Mechanisms, detection, and relevance of protein acetylation in prokaryotes. mBio 10:e02708-18. doi:10.1128/mBio.02708-18 30967470 PMC6456759

[45] Ouidir T , Jarnier F , Cosette P , Jouenne T , Hardouin J . 2015. Characterization of N-terminal protein modifications in Pseudomonas aeruginosa PA14. J Proteomics 114:214–225. doi:10.1016/j.jprot.2014.11.006 25464366

[46] Jones JD , O’Connor CD . 2011. Protein acetylation in prokaryotes. Proteomics 11:3012–3022. doi:10.1002/pmic.201000812 21674803

[47] Vetting MW , Bareich DC , Yu M , Blanchard JS . 2008. Crystal structure of RimI from Salmonella typhimurium LT2, the GNAT responsible for N(alpha)-acetylation of ribosomal protein S18. Protein Sci 17:1781–1790. doi:10.1110/ps.035899.108 18596200 PMC2548364

[48] Christensen DG , Meyer JG , Baumgartner JT , D’Souza AK , Nelson WC , Payne SH , Kuhn ML , Schilling B , Wolfe AJ . 2018. Identification of novel protein lysine acetyltransferases in Escherichia coli. mBio 9:e01905-18. doi:10.1128/mBio.01905-18 PMC6199490 30352934

[49] Pathak D , Bhat AH , Sapehia V , Rai J , Rao A . 2016. Biochemical evidence for relaxed substrate specificity of Nalpha-acetyltransferase (RV3420C/RimI) of Mycobacterium tuberculosis. Sci Rep 6:28892. doi:10.1038/srep28892 27353550 PMC4926160

[50] Yoshikawa A , Isono S , Sheback A , Isono K . 1987. Cloning and nucleotide sequencing of the genes rimI and rimJ which encode enzymes acetylating ribosomal proteins S18 and S5 of Escherichia coli K12. Mol Gen Genet 209:481–488. doi:10.1007/BF00331153 2828880

[51] Isono K , Isono S . 1980. Ribosomal protein modification in Escherichia coli. II. studies of a mutant lacking the N-terminal acetylation of protein S18. Mol Gen Genet 177:645–651. doi:10.1007/BF00272675 6991870

[52] Zhao Y. , Sun L , Champion MM , Knierman MD , Dovichi NJ . 2014. Capillary zone electrophoresis-electrospray ionization-tandem mass spectrometry for top-down characterization of the Mycobacterium marinum secretome. Anal Chem 86:4873–4878. doi:10.1021/ac500092q 24725189 PMC4033641

[53] Zhao Y , Riley NM , Sun L , Hebert AS , Yan X , Westphall MS , Rush MJP , Zhu G , Champion MM , Mba Medie F , Champion PAD , Coon JJ , Dovichi NJ . 2015. Coupling capillary zone electrophoresis with electron transfer dissociation and activated ion electron transfer dissociation for top-down proteomics. Anal Chem 87:5422–5429. doi:10.1021/acs.analchem.5b00883 25893372 PMC4439324

[54] Reyna C , Mba Medie F , Champion MM , Champion PA . 2016. Rational engineering of a virulence gene from Mycobacterium tuberculosis facilitates proteomic analysis of a natural protein N-terminus. Sci Rep 6:33265. doi:10.1038/srep33265 27625110 PMC5021934

[55] Buehl CJ , Deng X , Liu M , McAndrew MJ , Hovde S , Xu X , Kuo M-H . 2014. Resolving acetylated and phosphorylated proteins by neutral urea Triton-polyacrylamide gel electrophoresis: nut-page. Biotechniques 57:72–80. doi:10.2144/000114197 25109292 PMC4142444

[56] Champion PAD , Stanley SA , Champion MM , Brown EJ , Cox JS . 2006. C-terminal signal sequence promotes virulence factor secretion in Mycobacterium tuberculosis *.* Science 313:1632–1636. doi:10.1126/science.1131167 16973880

[57] Champion PAD , Champion MM , Manzanillo P , Cox JS . 2009. ESX-1 secreted virulence factors are recognized by multiple cytosolic AAA ATPases in pathogenic mycobacteria. Mol Microbiol 73:950–962. doi:10.1111/j.1365-2958.2009.06821.x 19682254 PMC3023814

[58] Champion MM , Williams EA , Kennedy GM , Champion PA . 2012. Direct detection of bacterial protein secretion using whole colony proteomics. Mol Cell Proteomics 11:596–604. doi:10.1074/mcp.M112.017533 22580590 PMC3434784

[59] Mba Medie F , Champion MM , Williams EA , Champion PAD . 2014. Homeostasis of N-alpha terminal acetylation of EsxA correlates with virulence in Mycobacterium marinum. Infect Immun 82:4572–4586. doi:10.1128/IAI.02153-14 25135684 PMC4249322

[60] Errey JC , Blanchard JS . 2005. Functional characterization of a novel ArgA from Mycobacterium tuberculosis. J Bacteriol 187:3039–3044. doi:10.1128/JB.187.9.3039-3044.2005 15838030 PMC1082834

[61] Ou J , Liu H , Nirala NK , Stukalov A , Acharya U , Green MR , Zhu LJ . 2020. DagLogo: an R/Bioconductor package for identifying and visualizing differential amino acid group usage in proteomics data. PLoS One 15:e0242030. doi:10.1371/journal.pone.0242030 33156866 PMC7647101

[62] Hartigan JA , Wong MA . 1979. A K-means clustering algorithm. J R Stat Soc C: Appl Stat 28:100–108. doi:10.2307/2346830

[63] Kapopoulou A , Lew JM , Cole ST . 2011. The MycoBrowser portal: a comprehensive and manually annotated resource for mycobacterial genomes. Tuberculosis (Edinb) 91:8–13. doi:10.1016/j.tube.2010.09.006 20980200

[64] Renshaw PS , Panagiotidou P , Whelan A , Gordon SV , Hewinson RG , Williamson RA , Carr MD . 2002. Conclusive evidence that the major T-cell antigens of the Mycobacterium tuberculosis complex ESAT-6 and CFP-10 form a tight, 1:1 complex and characterization of the structural properties of ESAT-6, CFP-10, and the ESAT-6*CFP-10 complex. Implications for pathogenesis and virulence. J Biol Chem 277:21598–21603. doi:10.1074/jbc.M201625200 11940590

[65] Bosserman RE , Nguyen TT , Sanchez KG , Chirakos AE , Ferrell MJ , Thompson CR , Champion MM , Abramovitch RB , Champion PA . 2017. WhiB6 regulation of ESX-1 gene expression is controlled by a negative feedback loop in Mycobacterium marinum. Proc Natl Acad Sci U S A 114:E10772–E10781. doi:10.1073/pnas.1710167114 29180415 PMC5740670

[66] Gottlieb L , Marmorstein R . 2019. Biochemical and structural analysis of N-terminal acetyltransferases. Methods Enzymol 626:271–299. doi:10.1016/bs.mie.2019.07.016 31606079 PMC6884420

[67] Champion MM , Williams EA , Pinapati RS , Champion PA . 2014. Correlation of phenotypic profiles using targeted proteomics identifies mycobacterial Esx-1 substrates. J Proteome Res 13:5151–5164. doi:10.1021/pr500484w 25106450 PMC4227905

[68] King CH , Mundayoor S , Crawford JT , Shinnick TM . 1993. Expression of contact-dependent cytolytic activity by Mycobacterium tuberculosis and isolation of the genomic locus that encodes the activity. Infect Immun 61:2708–2712. doi:10.1128/iai.61.6.2708-2712.1993 8500911 PMC280905

[69] Chirakos AE , Nicholson KR , Huffman A , Champion PA . 2020. Conserved ESX-1 substrates EspE and EspF are virulence factors that regulate gene expression. Infect Immun 88:e00289-20. doi:10.1128/IAI.00289-20 32900815 PMC7671884

[70] Bosserman RE , Nicholson KR , Champion MM , Champion PA . 2019. A new ESX-1 substrate in Mycobacterium marinum that is required for hemolysis but not host cell lysis. J Bacteriol 201:e00760-18. doi:10.1128/JB.00760-18 30833360 PMC6597391

[71] Sauer JD , Witte CE , Zemansky J , Hanson B , Lauer P , Portnoy DA . 2010. Listeria monocytogenes triggers AIM2-mediated pyroptosis upon infrequent bacteriolysis in the macrophage cytosol. Cell Host Microbe 7:412–419. doi:10.1016/j.chom.2010.04.004 20417169 PMC2947455

[72] Mittal E , Skowyra ML , Uwase G , Tinaztepe E , Mehra A , Köster S , Hanson PI , Philips JA . 2018. Mycobacterium tuberculosis type VII secretion system effectors differentially impact the ESCRT endomembrane damage response. mBio 9:e01765–01718. doi:10.1128/mBio.01765-18 30482832 PMC6282207

[73] Stamm LM , Morisaki JH , Gao L-Y , Jeng RL , McDonald KL , Roth R , Takeshita S , Heuser J , Welch MD , Brown EJ . 2003. Mycobacterium marinum escapes from phagosomes and is propelled by actin-based motility. J Exp Med 198:1361–1368. doi:10.1084/jem.20031072 14597736 PMC2194249

[74] Kinhikar AG , Verma I , Chandra D , Singh KK , Weldingh K , Andersen P , Hsu T , Jacobs WR Jr , Laal S . 2010. Potential role for ESAT6 in dissemination of M. tuberculosis via human lung epithelial cells. Mol Microbiol 75:92–106. doi:10.1111/j.1365-2958.2009.06959.x 19906174 PMC2846543

[75] Pajuelo D , Tak U , Zhang L , Danilchanka O , Tischler AD , Niederweis M . 2021. Toxin secretion and trafficking by Mycobacterium tuberculosis. Nat Commun 12:6592. doi:10.1038/s41467-021-26925-1 34782620 PMC8593097

[76] McDougal CE , Sauer JD . 2018. Listeria monocytogenes: the impact of cell death on infection and immunity. Pathogens 7:8. doi:10.3390/pathogens7010008 29324702 PMC5874734

[77] Sanchez KG , Ferrell MJ , Chirakos AE , Nicholson KR , Abramovitch RB , Champion MM , Champion PA . 2020. EspM is a conserved transcription factor that regulates gene expression in response to the ESX-1 system. mBio 11:e02807-19. doi:10.1128/mBio.02807-19 32019792 PMC7002343

[78] Saelens JW , Sweeney MI , Viswanathan G , Xet-Mull AM , Jurcic Smith KL , Sisk DM , Hu DD , Cronin RM , Hughes EJ , Brewer WJ , Coers J , Champion MM , Champion PA , Lowe CB , Smith CM , Lee S , Stout JE , Tobin DM . 2022. An ancestral mycobacterial effector promotes dissemination of infection. Cell 185:4507–4525. doi:10.1016/j.cell.2022.10.019 36356582 PMC9691622

[79] Williams EA , Mba Medie F , Bosserman RE , Johnson BK , Reyna C , Ferrell MJ , Champion MM , Abramovitch RB , Champion PA . 2017. A nonsense mutation in Mycobacterium marinum that is suppressible by a novel mechanism. Infect Immun 85:e00653-16. doi:10.1128/IAI.00653-16 27789543 PMC5278160

