# Supplementary material for: An N-acetyltransferase required for ESAT-6 N-terminal acetylation and virulence in Mycobacterium marinum
Source: mBio. 2023 Sep 29;14(5):e00987-23. doi: 10.1128/mbio.00987-23 (PMC10653941; doi:10.1128/mbio.00987-23)
Supplement: Supplemental Material — Figures S1–S8, Tables S1 and S2, supplemental methods, data set caption, and supplemental references. [file mbio.00987-23-s0001.pdf]

Supplementary Material for:

An N-acetyltransferase required for ESAT-6 N-terminal acetylation and virulence in *Mycobacterium marinum.*

Owen A. Collars, Bradley S. Jones, Daniel D. Hu, Simon D. Weaver, Taylor A. Sherman, Matthew M. Champion, Patricia A. Champion

Contents:

Figure S1. **Clustal alignment of the EsxA proteins from *M. marinum* and *M. tuberculosis.***

Figure S2. **Confirmation of *M. marinum* strains**

Figure S3**. Growth curve for Δ*MMAR1968 M. marinum* strains**

Figure S4. **A bacteriolysis reporter distinguishes between cytoplasmic and phagosomal *M. marinum* during macrophage infection**

Figure S5. **Representative images of macrophage cytolysis**

Figure S6. **TLC analysis of the relevant *M. marinum* strains**

Figure S7. **GFP expression in *M. marinum* strains grown under laboratory conditions**

Figure S8. **Representative images of spread assay**

Table S1. **Strains and plasmids used in this study**

Table S2. **Oligonucleotides used in this study**

Supplementary Materials and Methods

Supplementary Dataset

Supplementary References

```
EsxAMM          M-TEQQWNFAGIEAASSAIQGNVTSIHSLLDEGKQSLHKLAAAWGGSGSEAYRGVQQNWDS 60
EsxAMT          M-TEQQWNFAGIEAAASAIQGNVTSIHSLLDEGKQSLTKLAAAWGGSGSEAYQGVQQKWDA 60
                *-*************:********************* **************:****:**:

EsxAMM          TAQELNNSLQNLARTISEAGQAMSSTEGNVTGMFA 95
EsxAMT          TATELNNALQNLARTISEAGQAMASTEGNVTGMFA 95
                ** ****:***************:***********
```

Figure S1. Clustal alignment of the EsxA proteins from *M. marinum* and *M. tuberculosis.* EsxAMM: MMAR_5450 from *M. marinum,* EsxAMT: Rv3875 from *M. tuberculosis.* Sequences were obtained from Mycobrowser [1]. The N-terminal Met, green, is cleaved. The Thr at position 2, magenta, is the site of N-terminal acetylation. Shaded amino acids diverge between the two proteins.

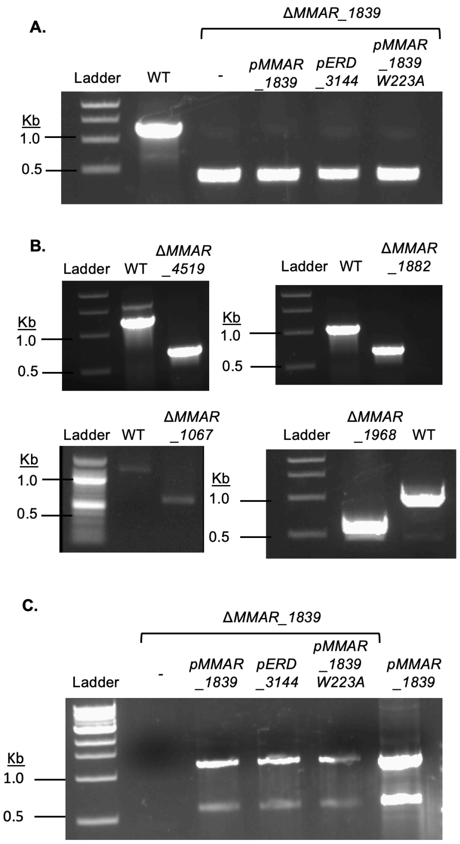

Figure S2. Confirmation of *M. marinum* strains. PCR confirming **A.** deletion of the *MMAR_1839* gene from *M. marinum*. Size of products: WT= 1218 bp, Δ*MMAR_1839*= 363 bp. **B.** deletion of additional conserved predicted NAT genes. WT *MMAR_4519* = 1232 bp, Δ*MMAR_4519*= 578 bp, WT *MMAR_1882*= 1036 bp, Δ*MMAR_1882*= 565 bp, WT *MMAR_1067*= 1221 bp, Δ*MMAR_1067*= 555 bp, WT *MMAR_1968*= 1051 bp, Δ*MMAR_1968*= 526 bp. **C.** presence of the integrating expression plasmid for complementation in the Δ*MMAR_1839* strain. In the final lane, the pure plasmid was used as the template for the PCR reaction as a positive control. All PCR primers are listed in Table S2.

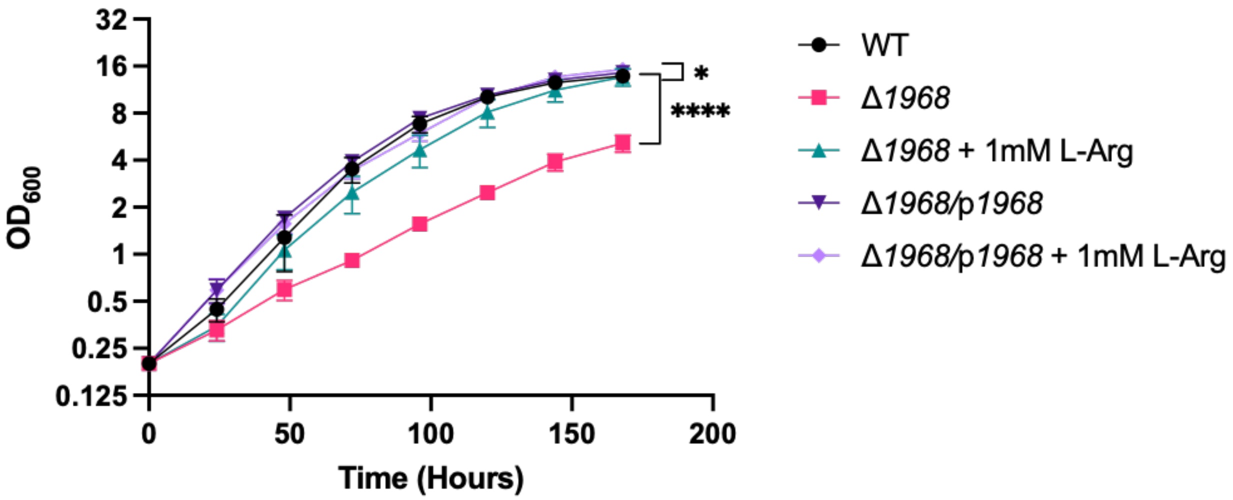

Figure S3. Growth curve for Δ*MMAR1968 M. marinum* strains. Growth curve of *M. marinum* strains grown in 7H9 + Tween in the presence of absence of 1mM L-Arginine. Data points are the average of three independent biological replicates, each measured in technical triplicate. Error bars represent the standard deviation. Significance was determined using a two-way ordinary ANOVA ($P<.0001$), **** $P<.0001$, Δ*1968* compared to WT, * $P=.0202$, Δ*1968*+ 1mM L-Arg compared to WT. The Δ*1968* and WT strains maintained the same significant from 72 hours through the end of the experiment. ($P<.0001$).

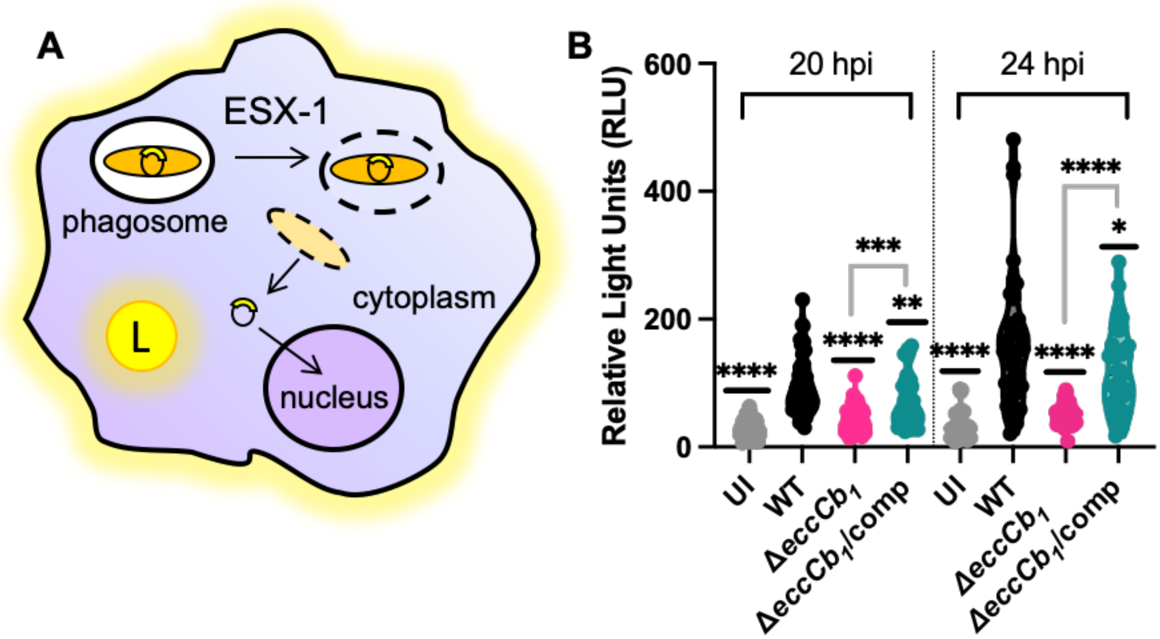

Figure S4: A bacteriolysis reporter distinguishes between cytoplasmic and phagosomal *M. marinum* during macrophage infection. A. Schematic of the bacteriolysis reporter, which was adapted from Sauer et al [2]. B. iIFNAR-/- macrophages were infected with *M. marinum* strains at an MOI of 20 for 20 or 24 hpi. All strains include the pTAS1 reporter plasmid. The data include three biological replicates. For each replicate, three independent infections are performed in independent wells. Data from three biological replicates (9 infections) are shown. Outliers were identified and removed using ROUT (Q = 0.5%). Significance was determined using a one way ordinary ANOVA (*P*<.0001) followed by a Tukey's comparison test comparing the strains and uninfected macrophages at each timepoint. **** *P*<.0001*,* For 20 hpi, *** *P*=.0002*,* ** *P*=.0048*,* For 24 hpi, * *P*=.0311*.*

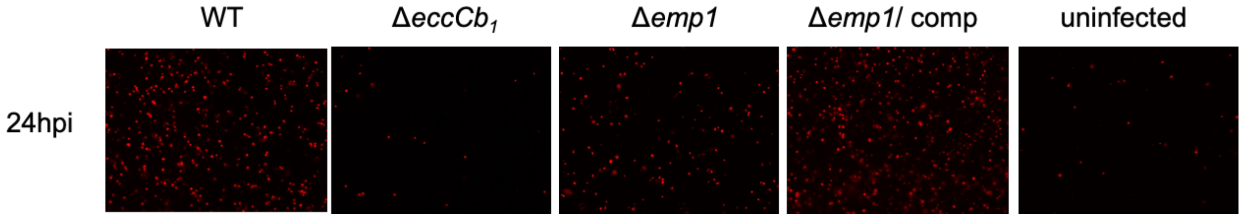

Figure S5. Representative images of macrophage cytolysis. RAW 264.7 cells were infected with *M. marinum* strains at an MOI of 5. Ethidium homodimer uptake was visualized at 24 hours post infection. The data are representative of at least 3 biological replicates with 10 fields selected for each infection. Counting the number of red cells per field resulted in the graph presented in Figure 4D.

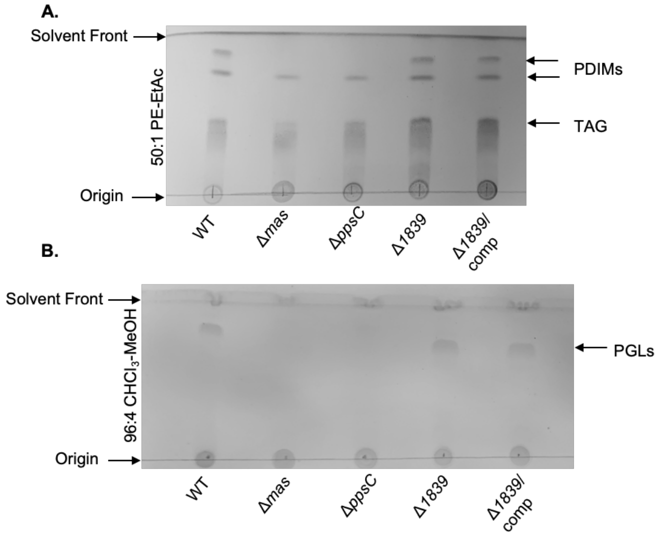

Figure S6. TLC analysis of the relevant *M. marinum* strains. Thin Layer Chromatography to confirm the presence of **A.** PDIMS and TAG, and **B**. PGLs. The WT strain is the positive control, the Δ*ppsC* and Δ*mas* strains are negative controls for all lipids measured. For all samples, 6µl of lipid extraction was spotted. The images are representative of at least three biological replicates.

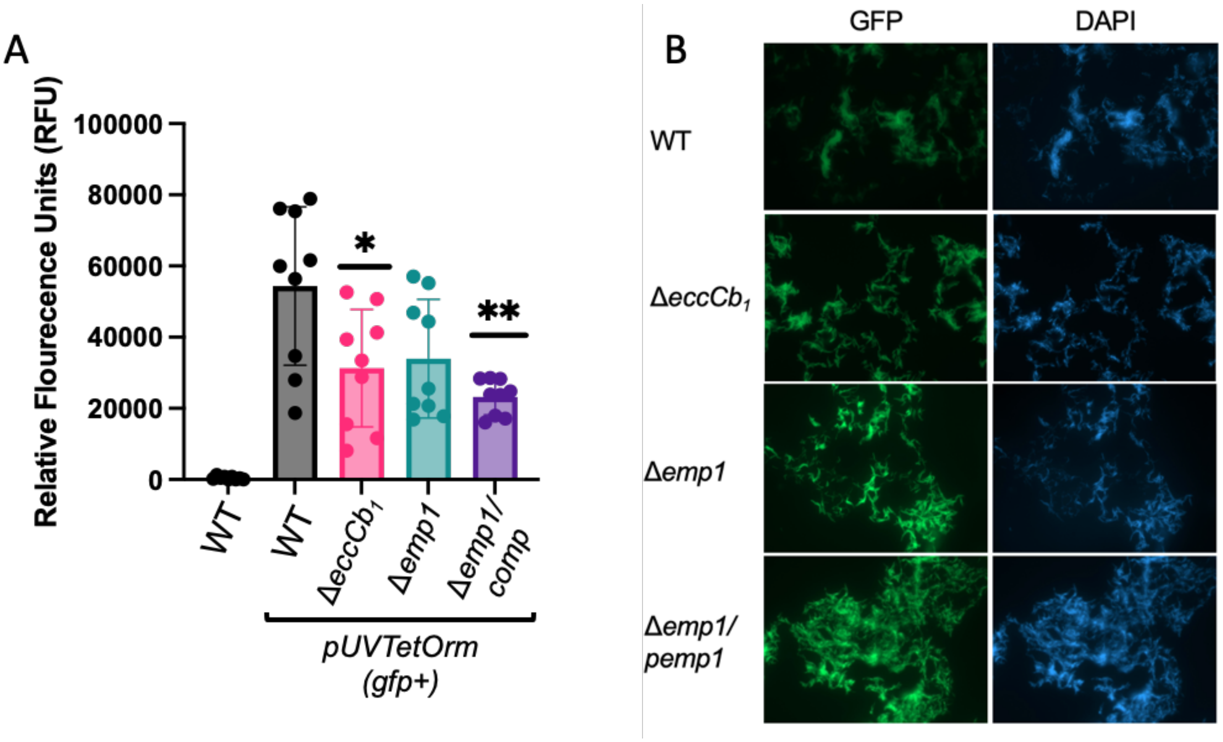

Figure S7. GFP expression in *M. marinum* strains grown under laboratory conditions. A. Measurement of GFP expression of *M. marinum* strains grown in 7H9 using a plate reader (population level). WT bacteria not expressing GFP were used a negative control for background fluorescence of the bacteria. B. Representative images of the same strains fixed to microscopy slides using 4% PFA and stained with DAPI. Images are 64x magnification in oil immersion. Individual bacteria can all be seen expressing GFP.

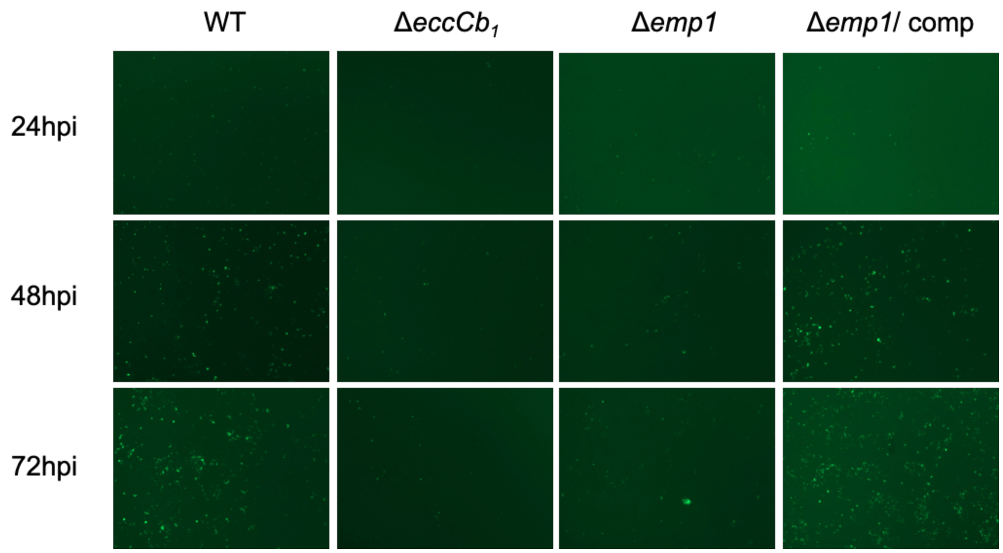

Figure S8. Representative images of spread assay. A RAW 264.7 monolayer was infected with *gfp* expressing *M. marinum* cells at an MOI of 1. Following treatment to kill external bacteria, the infected monolayer was overlaid with 0.8% agar. GFP signal was images at 24, 48 and 72 hours post infection. Counting GFP particles per field resulted in the violin plots presented in Figure 4E.

Table S1: Strains and plasmids used in this study

***M. marinum* Strains**		
**Name**	**Genotype**	**Reference**
M. marinum M strain	Wild-type strain; parental strain	ATCC BAA-535
ΔMMAR_1067	M strain with an unmarked in-frame deletion of the *MMAR_1067* gene	This study
ΔMMAR_1968	M strain with an unmarked in-frame deletion of the *MMAR_1968* gene	This study
ΔMMAR_1968/ pMMAR_1968	*ΔMMAR_1968* with the p*MMAR_1968* plasmid integrated at the *attB* site	This study
ΔMMAR_4519	M strain with an unmarked in-frame deletion of the *MMAR_4519* gene	This study
ΔMMAR_1839	M strain with an unmarked in-frame deletion of the *MMAR_1839* gene (named *erp1*)	This study
ΔMMAR_1882	M strain with an unmarked in-frame deletion of the *MMAR_1882* gene	This study
ΔesxBA	M with a deletion of the *esxBA* operon	[3]
ΔeccCb$_1$	M with a deletion of the *eccCb*$_1$ gene	[4]
ΔeccCb$_1$*/ peccCb*$_1$	*ΔeccCb*$_1$ with the p*eccCb1* plasmid integrated at the *attB* site.	[4]
Δemp1/ pemp1	Δ*MMAR_1839* with the p*MMAR_1839* plasmid integrated at the *attB* site	This study
Δemp1/ pERD_3144	Δ*MMAR_1839* with the p*ERD_3144* plasmid integrated at the *attB* site	This study
Δerp1/ perp1W223A	Δ*MMAR_1839* with the p*MMAR_1839W223A* plasmid integrated at the *attB* site	This study
ΔppsC	M strain with an unmarked in-frame deletion of the *ppsC* gene.	This study
Δmas	M strain with an unmarked in-frame deletion of the *mas* gene.	This study
WT / pTAS1	M strain with the pTAS1 plasmid.	This study
ΔeccCb$_1$ / pTAS1	*ΔeccCb*$_1$ with the pTAS1 plasmid.	This study
ΔeccCb$_1$ /*peccCb*$_1$/pTAS1	*ΔeccCb*$_1$ with the p*eccCb1* plasmid integrated at the *attB* site and the pTAS1 plasmid.	This study
Δemp1/ pTAS1	*Δemp1* with the pTAS1 plasmid.	This study
Δemp1/ pemp1/ pTAS1	*Δemp1* with the p*emp1* plasmid integrated at the *attB* site and the pTAS1 plasmid.	This study
Δemp1/ pUV15*tet*ORm	*ΔMMAR_1839* with the episomal pUV15*tet*ORm plasmid.	This study

Δemp1/ pemp1, pUV15*tet*ORm	*ΔMMAR_1839/pMMAR_1839* with the episomal pUV15*tet*ORm plasmid.	This study
**Plasmids**		
p2NIL	parental suicide vector for allelic exchange, Kan^R, Amp^R	[5], Addgene plasmid #20188
pGOAL19	marker cassette for allelic exchange, ampR, Hyg^R, *lacZ*+ *sacB*	[5], Addgene plasmid #20190
p2NILΔ*1067*GOAL	Allelic exchange plasmid to generate the Δ*MMAR_1067* strain, contains *M. marinum* flanking regions (NC010612.1: 1289146...1290701;1291328…1292886), p2NIL backbone with the GOAL19 marker cassette	This study
p2NILΔ*1968*GOAL	Allelic exchange plasmid to generate the Δ*MMAR_1968* strain, contains *M. marinum* flanking regions (NC010612.1: 2387011...2388617;2389075...2390645), p2NIL backbone with the GOAL19 marker cassette	This study
p2NILΔ*4519*GOAL	Allelic exchange plasmid to generate the Δ*MMAR_4519* strain, contains *M. marinum* flanking regions (NC010612.1: 5547155...5548785;5549330...5550919), p2NIL backbone with the GOAL19 marker cassette	This study
p2NILΔ*1839*GOAL	Allelic exchange plasmid to generate the Δ*MMAR_1839* strain, contains *M. marinum* flanking regions (NC010612.1: 2240745...2239051;2239899...2241609), p2NIL backbone with the GOAL19 marker cassette	This study
p2NILΔ*1882*GOAL	Allelic exchange plasmid to generate the Δ*MMAR_1882* strain, contains *M. marinum* flanking regions (NC010612.1: 2292737...2294457;2294823...2296515), p2NIL backbone with the GOAL19 marker cassette	This study
p2NILΔ*ppsC*GOAL	Allelic exchange plasmid to generate the Δ*ppsC* strain, contains *M. marinum* flanking regions (NC010612.1: 2156917...2158284;2159387...2160487), p2NIL backbone with the GOAL19 marker cassette	This study
p2NILΔ*masGOAL*	Allelic exchange plasmid to generate the Δ*mas* strain, contains *M. marinum* flanking regions (NC010612.1: 2132139...22133557;2139863...2141313),	This study

	p2NIL backbone with the GOAL19 marker cassette	
p*eccCb*$_1$	*eccCb*$_1$ (MMAR_5456) expressed behind the mycobacterial optimal promoter(p_{mop}), pMH406 backbone; Hyg^R, parental plasmid for expression plasmids in this study	[4]
p*emp1*	p*MMAR_1839* expressed behind the mycobacterial optimal promoter(p_{mop}), integrated at *attB*	This study
p*emp1W233A*	expressed behind the mycobacterial optimal promoter(p_{mop}), integrated at *attB;* W residue at position 223 changed to an A	This study
p*ERD_3144*	p*ERD_3144* expressed behind the mycobacterial optimal promoter(p_{mop}), integrated at *attB*	This study
p*MMAR_1968*	*MMAR_1968* expressed behind the mycobacterial optimal promoter (p_{mop}), integrated at *attB*.	This study
pUV15*tet*ORm	*gfp* expressed behind the Tet-ON promoter Hyg^R, Kan^R, episomal	[6]
pBHE573	*luc*+ gene expressed behind CMV enhancer, immediate early promoter, Kan^R, episomal	[2]
pMV261Kan	Parental plasmid with the P_{hsp60} promoter from BCG*;* Kan^R, episomal	[7]
pTAS1	*luc*+ gene expressed behind CMV enhancer, immediate early promoter, Kan^R, episomal	This study

Table S2: Oligonucleotides used in this study

Name	Sequence (5'-3')	Reference and Application
OMF241	CGTGGTGTCACGCTCGTGGTGTCCATGCTGGCGTACC	PCR primers for amplifying flanking regions of *MMAR_1067* for deletion, this study
OMF242	GCAGCCTTAAGGTGGTCGTGGTCTGTCATGGC	
OMF243	GACCACCTTAAGGCTGCCAACCCGGCCTGATCC	
OMF244	ACGCAGTCAGGCACCGTCACGTTGTTCGAAGTCGATCAGCCAAAGG	
OMF251	CGTGGTGTCACGCTCGTCGTTGTTGTACTCGACTGCCTTGG	PCR primers for amplifying flanking regions of *MMAR_1968* for deletion, this study
OMF252	GATGTTCTTAAGCCGGAAATCCTGTTGACTTTCG	
OMF253	TCCGGCTTAAGAACATCTTGGGCAACACCCG	
OMF254	ACGCAGTCAGGCACCGTTGGTACTCGACCGTGTCGTCG	
OMF259	CGTGGTGTCACGCTCGTGGTGAAGTTCAGCTCACCGACGC	PCR primers for amplifying flanking regions of *MMAR_4519* for deletion, this study
OMF260	CACCGACTTAAGCAGCGGACCGACATCCATCG	
OMF261	CGCTGCTTAAGTCGGTGGTCTCGACGCTGGTTCG	
OMF262	ACGCAGTCAGGCACCGTCGCTCACTTGTGAACCGAGTCTGCG	
OMF267	CGTGGTGTCACGCTCGTCGGTGACCTAACGGTCGAAGTGGGC	PCR primers for amplifying flanking regions of MMAR_1839 for deletion, this study
OMF268	GAAGGTCTTAAGCATGATGGGCGGAGCCGACATC	
OMF269	TCATGCTTAAGACCTTCGCCACGGTGCTGCTCG	
OMF270	ACGCAGTCAGGCACCGTCGTCCACCATCTTCGGGCTGATCG	
OMF275	CGTGGTGTCACGCTCGTCAGCTTAGAGGCGATCACCGAAGC	PCR primers for amplifying flanking regions of MMAR_1882 for deletion, this study
OMF276	GGAATCTTAAGGGTTGGTACGTCAAGGTCTTTGGCC	
OMF277	CCAACCCTTAAGATTCCGCACCTGCCCATGTTGC	
OMF278	ACGCAGTCAGGCACCGTGTGGCCCGTCCATCGGTGAGC	
OGC3	CCGGTCTGGAATCGTCAATC	*ΔMMAR_1067* confirmation, this study
OGC4	CGCTGGGTCAGATTGGGATG	
OGC5	ATTCGGTGGCCATGCCGTAG	*ΔMMAR_1968* confirmation, this study
OGC6	TGGTGGTGTCCAGTCAGTTC	
OGC7	TGGGCAAACGCCAACCGATG	*ΔMMAR_4519* confirmation, this study
OGC8	GCATGGGTACCAGCACAAAC	
OGC9	CGTCAACGGTCCTGGTGAAG	Δ*MMAR_1839* confirmation, this study
OGC10	AGAACAGTGACGCCAAGGAG	
OGC11	TCAGCGAGTGCAGGTGATCC	*ΔMMAR_1839* confirmation, this study
OGC12	TGTTCGCCATCGCGCAAGAC	

OGC25	CAGATCCAGGGGGTTGCGGTTCATCCGGAGCGG	Site-directed mutagenesis primers for changing W to A at position 233 of MMAR_1839, changes from WT sequence underlined, this study
OGC26	CCGCTCCGGATGAACCGCAACCCCCTGGATCTG	
OMF630	ACTAGTCGGGACCGCTCAGGCGTCC	p*eccCb*$_1$ vector amplification for FAST cloning, [8].
OMF057	CATATGGCTGGACTCCTGAATTCTGCAGCTG	
OMF096	ACGAGCGTGACACCACGATGCC	p2NIL vector amplification for FAST cloning, [9]
OMF097	ACGGTGCCTGACTGCGTTAGCAATTTAACTG	
oew264	GTGGTGTCACGCTCGTTGGCCTCAAACGCGCATCAG	*ppsC*$_{ER}$ domain knockout, upstream [9]
oew265	CAAACCGCCCATGCCATTTACCACGGTGCGGCGAGATTCGAG	
oew266	CGCACCGTGGTAAATGGCATGGGCGGTTTGGGTTTC	*ppsC*$_{ER}$ domain knockout, downstream [9]
oew267	GCAGTCAGGCACCGTTTGCCGTTTCCAGCTCGCTTG	
oew268	AACGCTTTGTCCACCGACTG	Δ*ppsC*$_{ER}$ confirmation, [9]
oew269	CGAGAAGGTCAGCCACCAATC	
orb146	TGGTGTCACGCTCGTGCAGTGAAGAACGGAGAGTC	*mas* knockout, downstream, this study
orb147	TCATAACGGCGGATCCGTTG	
orb148	GGATCCGCCGTTATGAGGCAGGTTTTTGCATAAATC	*mas* knockout, upstream, this study
orb149	GCAGTCAGGCACCGTCGTTGTCGTTCTTGCCATTC	
orb159	ACTGGATTCAGCCGGTGGTG	Δ*mas* confirmation, this study
orb160	ATCCGGCTTGGCCTGGATTG	
OBJ222	GGCCGCGGTGATCATCAATATTGGCCATTAG	pMV261kan vector amplification for FastCloning, this study
OBJ423	GTCGCTTTGTTGGCTAGCTACCACATTTGTAGAG	
OBJ424	GCTAGCCAACAAAGCGACGTTGTG	pBHE573 insert amplification for FastCloning, this study
OBJ225	TGATCACCGCGGCCATGATG	
OMF102	GGCTTAAGTATAAGGAGGAAAACATATGACCGAAAGTCAACAGGATTTCC	*MMAR_1968* insert for p*MMAR_1968* complementation plasmid, this study
OMF103	CTCCGTTTAGAGAGGGGTTATACTAGTTAAAGCACCAACAGCATCCGG	

OBJ384	AGGAGTCCAGCCATCAGATGTCGGCTCCGCCC	*ERD_3144* insert for FastCloning, this study
OBJ385	GCCTGAGCGGTCCCGACTAGTCGCTCAGTCCAGCAACAC	

Supplementary Materials and Methods:

Maintenance of Bacterial Strains: All *M. marinum* strains were derived from the *M. marinum* M strain (ATCC BAA-535). *M. marinum* strains were maintained in Middlebrook 7H9 defined broth (Sigma-Aldrich, St Louis MO) supplemented with 0.5% glycerol and 0.1% Tween-80 (Fisher Scientific, Pittsburgh PA) or on Middlebrook 7H11 agar (Sigma-Aldrich) plates supplemented with 0.5% glycerol and 0.5% glucose at 30°C. When necessary, agar plates and broth were supplemented with 20 µg/mL kanamycin (IBI Scientific, Peosta, IA), 50 µg/mL hygromycin (EMD Millipore, Billerica, MA), or 60µg/mL X-gal (Millipore). *E. coli* DH5α strains were grown at 37°C in LB (Luria-Bertani) media (VWR). When necessary, antibiotics were added to media at the following concentrations: 50 µg/mL kanamycin, 200 µg/mL hygromycin, or 200 µg/mL ampicillin (Thermo Fisher, Waltham, MA).

***MMAR_1968* L-Arginine Supplemented Growth Kinetics:** Wild type, *ΔMMAR_1968*, and p*MMAR_1968 M. marinum* strains were initially grown in a 5ml volume of Middlebrook 7H9 define broth (Sigma-Aldrich, St Louis MO) supplemented with 0.5% glycerol and 0.1% Tween-80 until mid-log phase. Strains were diluted to an optical density (OD_{600}) of 0.2 in 7H9 defined broth supplemented with 0.2% Tyloxapol (Chem-Impex International 24162, Wood Dale, IL). Where indicated, *ΔMMAR_1968*, and p*MMAR_1968* cultures were supplemented with 1mM L-Arginine (Sigma-Aldrich A6969, St. Louis, MO). Bacterial growth was assessed via optical density (OD_{600}) every 24 hours for 5 consecutive days. Strains were grown in technical triplicate. For each strain, data is representative of at least three biological replicates.

Generation of *M. marinum* Strains and Plasmids: Genetic deletions were constructed using allelic exchange as previously described [8-10]. ~1500 base pairs upstream and downstream of genes of interest were amplified with primers found in Table S2, and introduced into the p2NIL vector (Addgene plasmid number 20188; a gift from Tanya Parish) by three-part FastCloning as previously described [11]. Plasmid concentrations were measured on a NanoDrop

instrument (Thermo Fisher). Resulting constructs were digested with PacI(NEB) and dephosphorylated with Antarctic phosphatase (NEB). Enzymes were heat-inactivated at 80°C. The pGOAL19 vector (Addgene plasmid number 20190; a gift from Tanya Parish) was digested with PacI (NEB), which was heat inactivated at 65°C. The resulting pGOAL product was ligated into the digested p2NIL plasmid. 3 µg of plasmid was irradiated with 0.1 J/cm2 UV light in a CL-1000 UV cross-linker (UVP) followed by electroporation into 500µl of electrocompetent *M. marinum* cells using a GenePulser XCell (Bio-Rad). Electrocompetent cells were prepared exactly as described previously [12]. Transformed cells were recovered in 7H9 supplemented with 0.1% Tween 80 at 30°C overnight, spun down, resuspended in 200µL of the same media and plated on 7H11 agar (Sigma) supplemented with Kan, 60 µg/ml 5-bromo-4-chloro-3-indolyl-D-galactopyranoside (X-Gal), and oleic acid-albumin-dextrose-catalase (OADC). The resulting merodiploids were cultured in 7H9 broth, and then grown on 7H11 agar supplemented with OADC, 60 µg/ml X-Gal and 2% sucrose. White colonies were picked and cultured in 3 ml of 7H9 broth with 0.1% Tween-80. After approximately 5 days of growth, 500µl of culture were collected and lysed by three 30 second pulses on a mini bead beater (Biospec Products) followed by a 10-minute centrifugation to remove cell lysis products. 1µl of lysate was used in 10µl PCR reactions to confirm the genotype of each strain. The resulting PCR products were separated on TAE agarose gels stained with ethidium bromide (VWR) and imaged using a Gel Doc EZ imager (BioRad) and Image Lab software (BioRad). Plasmids and primers used for generation of knock-out strains can be found in Tables S1 and S2.

All complementation plasmids were generated by restriction digest using NdeI/SpeI to isolate each gene from *pUC19* plasmids containing genes of interest, followed by ligation of gene inserts into pMV406 vector. All complementation plasmids were confirmed using targeted Sanger DNA sequencing at the Notre Dame Genomics and Bioinformatics Facility.

The pTAS1 reporter plasmid was generated by FastCloning using primers found in Table S2 to amplify the insert sequence containing the *luc*+ gene, the CMV enhancer/immediate early

promoter, the chimeric intron region, and SV40 late polyadenylation signal from plasmid pBHE573 [2]. The pBHE573 was a gift of J.D. Sauer. The pMV261Kan vector was amplified with primers found in Table S2. The insert sequence was ligated into the amplified pMV261Kan vector resulting in the luciferase reporter plasmid, pTAS1. The plasmid was confirmed using whole plasmid sequencing by plasmidsaurus.

Site-Directed Mutagenesis (SDM): To change the Tryptophan at amino acid position 223 to an Alanine in the *emp1* gene, SDM was performed according to the instructions from the Quikchange II Site Directed Mutagenesis Kit (Agilent) [13-15]. The primers are listed in Table S2.

Protein Preparation and Analysis: Secretion Assays. ESX-1 protein secretion assays were performed as described previously [16]. Briefly, *M. marinum* strains were grown in 7H9 media, diluted to an OD_{600} of 0.8 in Sauton's Broth and grown for 48 hours. *M. marinum* was collected using centrifugation. The resulting supernatant was filtered through 0.2μm Nalgene Stericups with polyethersulfone (PES) filters and concentrated by ultrafiltration in a 3,000-molecular-weight-cutoff Amicon filter (Millipore). Cell- associated proteins were extracted from cells by lysis in PBS with a Biospec Mini-BeadBeater-24. Protein concentrations for cell-associated and secreted protein fractions were determined using the Pierce MicroBCA kit (Thermo Scientific).

NUT-PAGE: Neutral Urea Triton Polyacrylamide Gel Electrophoresis were performed as described in [17]**.** NUT-PAGE resolving gels were made with 6M Urea, 0.5M MOPs pH7, 4% Triton, 15% Polyacrylamide, 0.027% (w/v) APS, 0.13% (v/v) TEMED. Stacking gels were composed of 6M Urea, 9% Acrylamide, 100mM MOPs, 0.37% Triton-X, 0.06% (w/v) APS, 0.3% (v/v) TEMED. Running buffer was composed of 100mM Imidazole and 40mM MOPs pH7.10μg of protein were run in each lane for analysis. Gels were run for 1200V*hr, or 100V over the course of 12 hours.

Western Blotting. Unless otherwise noted, 10μg of protein were loaded onto 4-20% TGX-Gradient Gels (BioRad) for western blot analysis. All antibodies were diluted in 5% nonfat dry milk

in PBS with 0.1% Tween 20. RNAP polymerase subunit β (RNAP-β, ab12087; Abcam) was diluted 1:20,000. The monoclonal ESAT-6 (EsxA, Thermo Fisher) 1:500 or polyclonal ESAT-6 antibody, which was produced by Genscript, against the TEQQNFAGIEAAC peptide epitope, was used at 1:1,000. The AcEsxA polyclonal antibody was produced by Genscript against the Acetyl-TEQQNFAGIEAAC peptide epitope, and was used at 1:1,000. The following reagents were obtained through BEI Resources, NIAID, NIH: Polyclonal Anti-Mycobacterium tuberculosis CFP10 (Gene Rv3874) (antiserum, Rabbit), NR-13801 was used at 1:5,000; polyclonal anti-*Mycobacterium tuberculosis* Mpt32 (gene Rv1860, antiserum, rabbit) NR-13807, was used at 1:20,000. Horseradish peroxidase (HRP)-conjugated goat anti-mouse immunoglobulin secondary antibody (Bio-Rad) was used at 1:5,000 for detection of anti-RNAPβ and anti-EsxA (Thermo Fisher). HRP-conjugated goat anti-rabbit IgG secondary antibody (Bio-Rad) was used at a dilution of 1:5,000 for detection with all antibodies.

Dot Blot: 20µg of peptide were suspended in sterile water and applied to an Odyssey® nitrocellulose membrane and allowed to dry. Once completely dry the membranes were used for western blotting as follows. The membrane was blocked using Blocking Buffer for Fluorescent Western Blotting (Rockland). The αEsxA and αAc-EsxA primary antibodies were diluted together in blocking buffer at 1:500 and 1:1,000, respectively. The following secondary antibodies were used simultaneously at the following dilutions: A 1:5,000 dilution of Goat Anti-Mouse IgG (H+L) DyLight™ 800 Conjugated (Thermo Scientific) and 1:20,000 dilution of Goat Anti-Rabbit IgG (H+L) DyLight™ 680 Conjugated (Thermo Scientific).

Hemolysis Assays. Hemolysis assays were performed as in Cronin et al [16]. Briefly, *M. marinum* strains were grown in Middlebrook 7H9 broth and 0.1% Tween-80 to mid-log phase. The number of bacterial cells were normalized to absorbance at an optical density of 600 nm (OD_{600}). *M. marinum* cells were washed with PBS and resuspended in 300µl PBS. Sheep red blood cells (sRBCs, Hardy Diagnostics, Santa Maria, CA) were diluted 1:10 in PBS and dead sRBCs were removed. sRBC were resuspended in 500µl PBS. Washed bacteria were mixed with 100µl of

sRBCs, collected by centrifugation, and incubated at 30 °C for 1.5 hours. Each sample was read in technical triplicate in a 96-well plate on a SpectraMax ABS plate reader (Molecular Devices, San Jose, CA) at an optical density of 405nm (OD_{405}). For all hemolysis assays, incubation of sRBCs with water represents a maximal lysis positive control. Incubation of sRBCs with PBS alone, or with the Δ*eccCb₁* *M. marinum* strain, serve as negative controls for lysis. Error bars represent standard deviations. For each strain, data is representative of at least three biological replicates.

Macrophage infections: *Cytolysis:* Cytolysis assays were performed similar to those in Bosserman et al [18]. The RAW 264.7 murine macrophage cell line (ATCC TIB-71) was maintained in DMEM (Gibco) supplemented with 10% heat-inactivated fetal bovine serum (FBS, Seradigm) and maintained at 37°C with 5% CO_2. RAW 264.7 cells were plated at a density of 5×10^5 cells per well in cell culture treated 24-well plates (Greiner Bio-One) for 24 hours. The monolayer was infected with 2.5×10^6 *M. marinum* cells for a multiplicity of infection (MOI) of 5. Cells were incubated for 2 hours at 37 °C with 5% CO_2 and treated with 100 µg/mL gentamycin (Research Products International, Mount Prospect, IL) for 4 hours at 37 °C to kill extracellular *M. marinum* cells. Monolayers were washed three times with PBS, and new media was added to each well. Macrophage staining was performed using Ethidium homodimer-1 and Calcein AM from the Live/Dead Viability/Cytotoxicity kit (Thermo Fisher Scientific, Waltham, MA) at 24 hours post-infection. Cells were imaged by Zeiss AxioObserver A1 inverted microscope using phase-contrast, rhodamine, and green fluorescent protein filters. At least three independent biological replicates, each in technical triplicate (3 wells), were performed for each strain. For each well, 10 image fields were captured. Dead cells were analyzed and counted using Image J as previously described [10].

Colony Forming Units (CFUs): For CFU infections we performed as previously published in [18], except an MOI of 0.2. The resulting *M. marinum* cells were collected at 2, 24, 48, 72 and 96 hpi and plated at a dilution of 1:1000 for enumeration on agar.

Bacterial spread assay: To ensure that the *M marinum* strains expressed GFP uniformly and to rule out differences in GFP levels across the *M. marinum* strains, we measured GFP levels using a plate reader based assay. Mycobacteria strains carrying the pUV15*tetO*Rm plasmid were grown to mid log phase and induced with 100ng/mL anhydrotetracycline for 24 hours. The bacteria were diluted to an OD_{600} of 1.0 in 1x phosphate buffered saline (PBS) and washed three times to remove excess tween 80. GFP expressing *M. marinum* strains, as well as the WT parental strain without the pUVTetORm plasmid, and a 1xPBS blank were loaded on a costar 96-well plate with a clear bottom and black sides. The assay was performed on three independent cultures of each strain. In each experiment, three wells were measured for all strains and controls using a BioTek synergy H1 microplate reader. The plate was shaken, then an OD_{600} reading was recorded, shaken again, and then excited at a wavelength of 488nm followed by a recording of emission at 535nm. OD_{600} values and fluorescent emissions were corrected by the respective values for the 1xPBS blank. The relative fluorescence units were calculated by dividing the fluorescence level by their respective OD_{600}.

The *M. marinum* strains expressing the pUVTetORm plasmid were also visualized as follows. Mycobacteria strains expressing GFP were grown in 7H9 to mid logarithmic growth phase (three days) and GFP was induced with the addition of 100ng/mL ATc for 24 hours. Cells were then washed three times with 1xPBS and 10μL of washed cells were applied to Superfrost/Plus microscope slides (Fisher Scientific). Slides were left out until dry and then fixed to slides using drops of 4% paraformaldehyde (PFA) (Electron Microscopy Sciences). Cells were left in contact with 4% PFA for twenty minutes, then 1xPBS was added dropwise to wash for twenty minutes. After washing, 0.02mg/mL of 4′,6-diamidino-2-phenylindole (DAPI) (Cell signaling technologies) in 1xPBS was added to the fixed cells for twenty minutes at room temperature in the dark. After removing the 1xPBS-DAPI mixture, cells were mounted with a Premium Cover Glass from Fisher Scientific using ProLong Gold antifade reagent (Invitrogen) and sealed with clear nail polish.

Slides were imaged using Zeiss AxioObserver A1 inverted microscope using DAPI and green fluorescent filters.

The cell to cell spread assay was adapted from [3, 19]. RAW 264.7 murine macrophage cell line was maintained in DMEM supplemented with 10% heat-inactivated FBS and maintained at 37°C with 5% CO_2. RAW 264.7 cells were plated at a density of 4×10^5 cells per well in cell culture treated 24-well plates for 24 hours. The monolayer was infected with *M. marinum* strains at a MOI of 1 and incubated for 2 hours at 37 °C with 5% CO_2 and treated with 100 µg/mL gentamycin for 2 hours at 37 °C to kill extracellular *M. marinum* cells. Monolayers were washed three times with PBS, and overlaid with 0.8% agarose to prevent monolayer movement. New media including 100ng/mL anhydrotetracycline (ATc, Acros Organics) was added to each well to induce *gfp* expression in *M marinum*. Media was changed at 48 hours post-infection. Each infection was performed in biological quadruplicate, each in technical triplicate. 10 representative photos were taken at 48- and 72-hours post-infection for each well. Only three photos were taken in the first and second biological replicates of 24-hours post-infection, while the other two replicates had 10 photos taken per well. Cells were imaged by Zeiss AxioObserver A1 inverted microscope using green fluorescent protein filters at 10X magnification. Bacterial cells were counted using Image J as previously described [10]. ROUT analysis [20] in GraphPad Prism with a Q value of 0.5% was used to detect and remove outliers from bacterial count data.

<u>*Bacteriolysis:*</u> Intracellular bacteriolysis assays were performed as in Sauer et al [2]. Briefly, nonstimulated bone marrow-derived iIFNAR-/- murine macrophages (gifted from Dr. John-Damien Sauer) were maintained in Immortalized Macrophage Media (iMM). iMM is RPMI Medium 1640 (Gibco) supplemented with 10% heat-inactivated fetal bovine serum (FBS, Seradigm), 1% sodium pyruvate (Gibco), 1% L-glutamine (Gibco), and 0.1% β-mercaptoethanol (Gibco). Macrophages were maintained at 37°C with 5% CO_2. Macrophages were plated at 5×10^5 cells per well in cell culture treated 24-well plates (Greiner Bio-One) for 24 hours. The monolayer was infected with 1×10^7 *M. marinum* containing the pTAS1, MOI of 20. The infection proceeded for 2

hours at 37°C with 5% CO_2 and treated with 100 µg/ml gentamycin (Research Products International, Mount Prospect, IL) for 4 hours at 37°C with 5% CO_2 to kill extracellular *M. marinum* cells. Monolayers were washed with PBS, and fresh iMM was added to each well. At 20 and 24 hours post-infection the iMM was removed from each well. 300 µl of TNT lysis buffer (20mM Tris Base, 100mM NaCl, 1% Triton X-100 [pH 8]) was added to each well and incubated for 10 minutes at room temperature to lyse cells. Cell lysates were transferred in triplicate from each well to an opaque 96-well plate (Greiner Bio-One). Firefly Luciferase Substrate was added to each well and luciferase activity was measured (Bio-Tek H1 Synergy Multimode Plate Reader).

Thin layer chromatography (TLC): Total lipid extraction from *M. marinum* followed by TLC was performed as in [4] with the following changes. *M. marinum* strains were grown to saturation in 7H9 broth. *M. marinum* were diluted to an OD_{600} of 0.05, grown in 50ml of 7H9 broth for 48 hours and then collected and washed 3 times in PBS. Following total lipid extraction, the samples were evaporated in a fume hood. 6µl of lipids were migrated via capillary action for approximately 15 minutes. PDIM lipids were migrated 3 times sequentially.

Mass Spectrometry

Reagents and Equipment: LC-MS pure reagents (water, chloroform, acetonitrile, and methanol) were purchased from J.T. Baker (Radnor, PA). Tris(2-carboxyethyl)phosphine (TCEP) was purchased from TCI Chemicals (Portland, OR). Iodoacetamide (IAA) was purchased from VWR (Radnor, PA). S-Traps were purchased from Protifi. Unless specified, all other reagents were purchased from Sigma-Aldrich (St. Louis, MO). Hydrophilic–lipophilic balance (HLB) solid phase extraction (SPE) cartridges (10mg/1ml) were purchased from Waters Corporation (Milford, MA). Vacuum concentrator used was a Genevac miVac from SP Scientific (Warminster, PA).

Label Free Quantification (LFQ): Sample preparation was performed similarly to as previously described [9, 18, 21]. 100µg of protein from each *M. marinum* lysate sample were extracted by acetone precipitation. Dried samples were resuspended in 10µL 100mM TEAB, 5µL

1M TEAB, and 25µL 20% SDS. Proteins were reduced by addition of 5µL 100mM TCEP and incubated for 10 minutes at 95˚C. Proteins were subsequently alkylated with the addition of 5µL 100mM IAA for 30 minutes in darkness. Samples were acidified with 5µL of 12% H_3PO_4, and flocculated with 350µL of 90/10 methanol/100mM TEAB. Samples were each loaded and spun through S-Trap 'Minis' to immobilize proteins on-filter. Bound protein was washed with two additions of 150µL 90/10 methanol/100mM TEAB and one addition of 150µL 1:1 methanol/chloroform. Filters were placed into new collection tubes, and 2µg of sequencing grade trypsin (Promega, Madison, WI) in 160µL, 100mM TEAB was added, to a 1:50 wt/wt enzyme-to-protein ratio. Samples were incubated for 4 hours at 37˚C. Following digestion, samples were spun down and eluted by two additions of 80µL 0.1% formic acid in water and one addition of 80µL 1:1 acetonitrile/water in 0.1% formic acid. Samples were dried on a vacuum concentrator for 20 minutes to reduce residual acetonitrile. Remaining sample was desalted with HLB SPE cartridges on a vacuum manifold according to manufacturer's instructions.

Samples were resuspended in 0.1% formic acid in water to 500ng/µL. Each sample had 1µL injected in triplicate into a Bruker nanoElute and timsTOF Pro MS system. A 90-minute 600nL/min gradient from 4-30% B (A= water + 0.1% formic acid, B= acetonitrile + 0.1 formic acid) was used on a 75µm x 100mm PepSep column with C_{18} ReproSil AQ stationary phase at 1.9µm particle size, 120 Å pore size. CaptiveSpray nano-ESI with a spray voltage of 1700V. MS was set to PASEF mode with a mass range of 100-1700 m/z, ion mobility range of 0.6-1.6 v^*s/cm^2, and ramp and accumulation times of 100ms. Each precursor consisted of 10 PASEF ramps for a cycle time of 1.17 seconds. Precursors were filtered to contain only charges from 2 to 5. MS/MS collision energy settings were set to ramp from 20eV at 0.6 ion mobility to 70eV at 1.6 ion mobility. Instrument tune parameters were set to default for proteomic studies with the following differences: quadrupole low mass set to 20 m/z, focus pre-TOF pre-pulse storage set to 5µs.

Peptide spectral mass matching and quantitative LFQ were performed on the raw data using PEAKS Online Xpro (build 1.4.2020-10-21_171258). A PEAKS Q analysis was performed

to include label-free quantitative and FDR was controlled at the protein and peptide levels to <1%. (LFQ) data [22]. The database searched against was an *M. marinum* M strain proteome FASTA file from Mycobrowser (Release 4, [1]). Fixed modification of carbamidomethylation of cysteines was set. Variable modifications were set: acetylation of protein N-terminus, deamidation of asparagine and glutamine, oxidation of methionine, conversion of glutamine and glutamic acid to pyroglutamic acid. Quantitation was normalized using the total ion current (TIC). All other search parameters were default unless specified. Raw and processed data are available through MassIVE and PrideDB ftp://MSV000091442@massive.ucsd.edu ftp://massive.ucsd.edu/MSV000091442/ Pride PXD040693.

Protein and peptide .csv files were exported from PEAKS, and the following analysis was performed using R. Each biological replicate had three strains: Wild type (WT), *ΔMMAR_1839,* and Complement (Comp), which each had three triplicate injections. Each peptide LFQ intensity was normalized to the protein RpoA LFQ value from its respective injection. For each biological replicate, the peptide was discarded if it was not observed in at least four observations from the six injections of WT and Comp (2 biological 3 technical replicates). Peptides were not considered if they did not start at the first or second canonical amino acid position of the protein. The average, standard deviation, and %RSD was calculated for each peptide for each strain. If there were not sufficient datapoints/degrees of freedom to calculate a standard deviation for a particular peptide/strain combination, the average %RSD for all other peptides in that peptide/strain combination was assigned. Peptides containing an N-terminal acetylation modification were separated from those that did not, ratios of average LFQ intensity for the *ΔMMAR_1839*:WT and Δ*MMAR_1839*: Δ*MMAR_1839/*complemented were calculated from the non-terminal peptides. Peptides within proteins with ratios ≥ one were discarded. Remaining N-terminally acetylated peptides were matched with the non-acetylated cognate, and a total LFQ intensity for that peptide was calculated by summing the values. If multiple peptide variants existed for any protein (e.g. resulting from a missed cleavage or other PTM), the intensities of all peptides were summed

together to obtain a total acetylated and non-acetylated LFQ value for each protein in the dataset. From these data % acetylation values were calculated by taking the ratio of acetylated LFQ intensity / total LFQ intensity for each protein. *k*-means clustering analysis was performed with each acetylated protein in the dataset. The variables considered for the clustering were the following LFQ intensity ratios: Δ*MMAR_1839*:Δ*MMAR_1839/*comp, Δ*MMAR_1839*:WT, and Δ*MMAR_1839/*comp:WT, and clustering was performed with the kmeans() function in R, after each variable was centered using the base scale() function in R to reduce the influence of outliers. The proteins were clustered into three groups, using 25 random starting points, and those proteins clustering with EsxA were identified as potential acetylation targets of MMAR_1839. Code for this analysis can be found on GitHub (https://github.com/Champion-Lab/ESXA_Acetylation) along with a list of data analysis steps.

MALDI: Whole colony MALDI was performed exactly as described in Champion et al. [12]. Briefly whole colonies from WT, Δ*eccCb*$_1$, Δ*MMAR_1839*, and the three complementation strains were picked and placed in a 0.45 µm spin filter (Costar) and briefly mixed with 100µl MS-grade water. Washate was collected by spinning and 1µl was spotted on a stainless steel MALDI target, dried and 1µl of a saturated solution of sinapinic acid in 50% acetonitrile/water was overlaid. MS spectra were acquired in linear mode on a Bruker Autoflex Extreme MALDI-TOF. A laser frequency of 500Hz was used and 10,000 spectra were summed with a mass range of 9,500 – 12,000 m/z. Mass calibration was performed using the $[M+1H]^{1+}$ and $[M+2H]^{2+}$ peaks from horse heart myoglobin spotted adjacent to the samples. MS spectra were exported as ASCII and imported into Prism Graphpad for visualization.

Bioinformatics: The k-means clustering, generation of the SeqLogos, IceLogos and heatmaps were generated using R. All of the relevant code can be found on Github at the following link: https://github.com/Champion-Lab/ESXA_Acetylation.

Statistical analysis was performed using Prism v. 9 as indicated in the figure legends.

Supplementary Dataset:
Tab S1A. Raw search results (.csv peptide/protein export from peptide-spectral mass matching, uploaded to MassIVE due to size restrictions, ftp://MSV000091442@massive.ucsd.edu).
Tab S1B. Trimmed Search Results Identical to SIA but contaminant/decoy proteins and unused columns have been removed.
Tab S1C. Bioreplicate Frequency. Trimmed, summed data for protein/peptide Termini for all N-terminally acetylated proteins observed across bioreplicates
Tab S1D. EsxA plot. Tabular data used to generate data in Figure 2
Tab S1E. Bioreplicate 3 cluster 3. Processed data as M&M and in Bioreplicate frequency used for k-means clustering in Figure 2. Shown are the predicted orthologs in *M. tuberculosis* H37Rv, protein product and functional categories derived from Mycobrowser[1].

Supplementary References

1. Kapopoulou, A., J.M. Lew, and S.T. Cole, *The MycoBrowser portal: a comprehensive and manually annotated resource for mycobacterial genomes.* Tuberculosis (Edinb), 2011. **91**(1): p. 8-13.
2. Sauer, J.D., et al., *Listeria monocytogenes triggers AIM2-mediated pyroptosis upon infrequent bacteriolysis in the macrophage cytosol.* Cell Host Microbe, 2010. **7**(5): p. 412-9.
3. Gao, L.Y., et al., *A mycobacterial virulence gene cluster extending RD1 is required for cytolysis, bacterial spreading and ESAT-6 secretion.* Mol Microbiol, 2004. **53**(6): p. 1677-93.
4. Williams, E.A., et al., *A Nonsense Mutation in Mycobacterium marinum That Is Suppressible by a Novel Mechanism.* Infect Immun, 2017. **85**(2).
5. Parish, T. and N.G. Stoker, *Use of a flexible cassette method to generate a double unmarked Mycobacterium tuberculosis tlyA plcABC mutant by gene replacement.* Microbiology, 2000. **146 (Pt 8)**: p. 1969-75.
6. Ehrt, S., et al., *Controlling gene expression in mycobacteria with anhydrotetracycline and Tet repressor.* Nucleic Acids Res, 2005. **33**(2): p. e21.
7. Stover, C.K., et al., *New use of BCG for recombinant vaccines.* Nature, 1991. **351**(6326): p. 456-60.
8. Sanchez, K.G., et al., *EspM Is a Conserved Transcription Factor That Regulates Gene Expression in Response to the ESX-1 System.* mBio, 2020. **11**(1).
9. Bosserman, R.E., et al., *WhiB6 regulation of ESX-1 gene expression is controlled by a negative feedback loop in Mycobacterium marinum.* Proc Natl Acad Sci U S A, 2017.
10. Chirakos, A.E., et al., *Conserved ESX-1 substrates EspE and EspF are virulence factors that regulate gene expression.* Infect Immun, 2020.
11. Li, C., et al., *FastCloning: a highly simplified, purification-free, sequence- and ligation-independent PCR cloning method.* BMC Biotechnol, 2011. **11**: p. 92.
12. Champion, M.M., et al., *Direct detection of bacterial protein secretion using whole colony proteomics.* Mol Cell Proteomics 2012. **11**: p. 596-604.
13. Sanchez, K.G., et al., *Functional Analysis of EspM, an ESX-1-Associated Transcription Factor in Mycobacterium marinum.* J Bacteriol, 2022: p. e0023322.
14. Champion, P.A., et al., *C-terminal signal sequence promotes virulence factor secretion in Mycobacterium tuberculosis.* Science, 2006. **313**(5793): p. 1632-6.
15. Champion, P.A., et al., *ESX-1 secreted virulence factors are recognized by multiple cytosolic AAA ATPases in pathogenic mycobacteria.* Mol Microbiol, 2009. **73**(5): p. 950-62.
16. Cronin, R.M., et al., *Proteo-genetic analysis reveals clear hierarchy of ESX-1 secretion in Mycobacterium marinum.* Proc Natl Acad Sci U S A, 2022. **119**(24): p. e2123100119.
17. Buehl, C.J., et al., *Resolving acetylated and phosphorylated proteins by neutral urea Triton-polyacrylamide gel electrophoresis: NUT-PAGE.* Biotechniques, 2014. **57**(2): p. 72-80.
18. Bosserman, R.E., et al., *A New ESX-1 Substrate in Mycobacterium marinum That Is Required for Hemolysis but Not Host Cell Lysis.* J Bacteriol, 2019. **201**(14).
19. Castro-Garza, J., et al., *Demonstration of spread by Mycobacterium tuberculosis bacilli in A549 epithelial cell monolayers.* FEMS Microbiology Letters, 2002. **212**(2): p. 145-149.
20. Motulsky, H.J. and R.E. Brown, *Detecting outliers when fitting data with nonlinear regression - a new method based on robust nonlinear regression and the false discovery rate.* BMC Bioinformatics, 2006. **7**: p. 123.
21. Saelens, J.W., et al., *An ancestral mycobacterial effector promotes dissemination of infection.* Cell, 2022.

22. Zhang, J., et al., *PEAKS DB: de novo sequencing assisted database search for sensitive and accurate peptide identification.* Mol Cell Proteomics, 2012. **11**(4): p. M111 010587.
